# Protease-Inhibitor Interaction Predictions: Lessons on the Complexity of Protein–Protein Interactions * [Fn FN2]

**DOI:** 10.1074/mcp.M116.065706

**Published:** 2017-04-06

**Authors:** Nikolaus Fortelny, Georgina S. Butler, Christopher M. Overall, Paul Pavlidis

**Affiliations:** From the ‡Department of Biochemistry and Molecular Biology;; §Michael Smith Laboratories;; ¶Centre for Blood Research;; ‖Department of Oral Biological and Medical Sciences, Faculty of Dentistry;; **Department of Psychiatry, University of British Columbia, Vancouver, British Columbia, Canada

## Abstract

Protein interactions shape proteome function and thus biology. Identification of protein interactions is a major goal in molecular biology, but biochemical methods, although improving, remain limited in coverage and accuracy. Whereas computational predictions can guide biochemical experiments, low validation rates of predictions remain a major limitation. Here, we investigated computational methods in the prediction of a specific type of interaction, the inhibitory interactions between proteases and their inhibitors. Proteases generate thousands of proteoforms that dynamically shape the functional state of proteomes. Despite the important regulatory role of proteases, knowledge of their inhibitors remains largely incomplete with the vast majority of proteases lacking an annotated inhibitor. To link inhibitors to their target proteases on a large scale, we applied computational methods to predict inhibitory interactions between proteases and their inhibitors based on complementary data, including coexpression, phylogenetic similarity, structural information, co-annotation, and colocalization, and also surveyed general protein interaction networks for potential inhibitory interactions. In testing nine predicted interactions biochemically, we validated the inhibition of kallikrein 5 by serpin B12. Despite the use of a wide array of complementary data, we found a high false positive rate of computational predictions in biochemical follow-up. Based on a protease-specific definition of true negatives derived from the biochemical classification of proteases and inhibitors, we analyzed prediction accuracy of individual features, thereby we identified feature-specific limitations, which also affected general protein interaction prediction methods. Interestingly, proteases were often not coexpressed with most of their functional inhibitors, contrary to what is commonly assumed and extrapolated predominantly from cell culture experiments. Predictions of inhibitory interactions were indeed more challenging than predictions of nonproteolytic and noninhibitory interactions. In summary, we describe a novel and well-defined but difficult protein interaction prediction task and thereby highlight limitations of computational interaction prediction methods.

Identification of protein interactions is an important goal in molecular biology yet one that remains difficult. Approaches such as yeast-2-hybrid, coimmunoprecipitation and newer experimental methods (1, 2) are highly productive and scalable. However, limited accuracy from false positives and coverage that is context dependent remain problematic (3, 4). Computational methods have been developed to predict protein–protein interactions, commonly linking together proteins on the basis of shared features such as patterns of conservation, expression, or annotations (5–8)—a version of guilt by association. A second class of approaches uses protein structural features to identify potential physical interaction interfaces (9). These approaches can be combined. However, their practical utility remains unclear. In the methods cited above, accuracy was estimated by cross-validation or by validating a small number of hand-picked test cases (5, 6). Estimates of the true efficacy of prediction methods in structured evaluations, such as those that exist for function prediction (critical assessment of protein function annotation algorithms (10)), structure prediction (critical assessment of protein structure prediction (11)), or for structural docking (critical assessment of prediction of interactions (12)), are lacking for protein interaction prediction methods. If computational predictions of interactions were sufficiently accurate, biochemical assays could be targeted more efficiently by focusing on predicted pairs (9), but to date, computational predictions do not appear to have played a major role in interaction discovery or prioritization (13). We hypothesized that studying a specific subset of protein interactions and combining computational prediction and biochemical validation will grant deeper insights into the pitfalls and state of the art for general protein interaction predictions.

We focused on the prediction of interactions between protease inhibitors and proteases—a problem that has not received specific attention to our knowledge—despite being characterized by covalent or low-*K_D_* noncovalent interactions (low nm or pm) and hence, in principle, being more tractable for identification than high-*K_D_* noncovalent, general protein–protein interactions. Previous cell culture and transcript analyses have suggested that known protease–inhibitor pairs are often coexpressed and coregulated (14, 15). It is therefore hypothesized that protease–inhibitor coexpression plays a major role in the regulation of the detrimental activities of a protease. Inverse protease–inhibitor coexpression is thought to amplify protease activity but has only been observed for relatively few protease–inhibitor pairs (16, 17). Overall, it is currently a common assumption that protease–inhibitor coexpression is evidence for an inhibitory interaction, but this concept has not been tested comprehensively.

Proteases are a critical component of the posttranslational regulatory machinery in cells and therefore promising drug targets. However, drug targeting of proteases has been hampered by complex protease biology that is often poorly understood. One aspect of this complexity is the organization of proteases in dense interaction networks of protease cleavage and interaction (18). Proteases regulate the activity of other proteases by direct cleavage or by cleaving their endogenous inhibitors, which in turn influences additional distal cleavage events. Thus, proteases can potentially indirectly influence the cleavage of substrates other than their direct substrates. We recently established a graph model of protease web interactions based on existing biochemical data that can be used to predict proteolytic pathways (19). However, the network is far from its full potential because cleavage and inhibition interaction data underlying the model are incomplete. This is mainly due to the lack of studies of proteases and inhibitors but also to the lack of uploading of existing data to community databases. Computational prediction could provide a means to accelerate the addition of interactions to this network. However, large-scale computational prediction efforts in protease interaction biology have been limited to the use of molecular features of proteases and their substrates to predict protease cleavage (20) and have largely ignored protease inhibition. Therefore, the whole realm of protease inhibition is underexplored, with 354 (∼80%) of 444 human proteases lacking annotated inhibitors and 13 (∼14%) of 94 inhibitors without any annotated targets (orphan inhibitors) in the MEROPS protease database (21). Proteases are regulated by multiple mechanisms other than inhibition such as autodegradation, reversible activation, substrate-induced activation, and other allosteric activators. However, protease inhibitors are often present in adjacent compartments to block and clear excess proteases that could rapidly and irreversibly cleave a large number of proteins. Protease inhibitors are therefore often secreted in the plasma or distal tissues to block proteases delivered by diffusion, secretion, or leakage from tissues to the circulation. Considering the key role of proteases in cell signaling pathways, identifying additional, physiologically relevant protease–inhibitor pairs would greatly benefit our understanding of protease biology.

Important questions in interaction prediction methods are which input data to use for predictions and how to evaluate performance (in contrast, the prediction algorithm used plays relatively little role (22)). To evaluate performance of a predictor, efficacy in separating predefined true positives (TP) ^1^ and true negative (TN) examples is measured. For example, in interaction prediction, if most true interacting proteins are coexpressed and noninteractors are not coexpressed, then coexpression is a good predictor of interaction. The better the separation of the two groups, the better the predictive performance. In general, TPs are readily found in biological databases, but the definition of TNs is a challenge, especially for weak interactions having low mm *K_D_*s, and more practically since a lack of interaction is rarely established and documented. Common approaches therefore use unlikely interactions as TNs, for example, random interactions (based on the assumption that true interactions are a small subset of all possible interactions) or interactions between proteins localized to different cellular compartments according to annotation (4). An advantage of the protease–inhibitor prediction task is the ability to define TP and TN inhibitions more accurately. Protease inhibitors are characterized by tight interactions with their cognate proteases, thus providing a clear separation between true and false interactors. Further, proteases and their inhibitors are organized into families based on their primary sequence and into clans based on the structure of their active site and reactive site, respectively (21). Families and clans of inhibitors can mostly be assigned specifically to one or two target protease classes. Thus, it is possible to define TN pairs, where the inhibitor cannot inhibit the protease based on known chemical and structural constraints. As examples, a serpin will not inhibit a metalloprotease, and a tissue inhibitor of metalloproteinases will neither inhibit a serine protease nor aspartate, threonine, or cysteine proteases. However, matrix metalloproteinases (MMPs) cleave and inactivate many serpins and so transiently are also interactors before peptide bond scission, albeit with a moderate *K_D_* (∼ *K_m_*) (18, 23). A further advantage of selecting this group of proteins in the analysis of prediction methods is the accuracy of biochemical testing of the predictions by measuring inhibition of the catalytic activity of the protease.

Here, we defined TP inhibitions (*n* = 294) as those inhibitions annotated in MEROPS (21). We defined TN inhibitions (*n* = 6,990) as enzymatically implausible inhibitor/protease pairs that are known not to be inhibitory. Using this gold standard, we evaluated the predictive power of common interaction prediction methodology to predict protease–inhibitor pairs in the protease web. Predictions were based on protein–protein interaction data, coannotation, coexpression, phylogenetic similarity, and colocalization as input data. Interestingly, we report that coexpression is surprisingly low for many functional protease–inhibitor pairs, contrary to what is commonly assumed. In particular, we employed 40 interaction predictors based on coexpression values derived from different input data and correlation metrics, all of which we found suffered from weak predictive power. Nonetheless, we predicted 270 protease–inhibitor pairs, examined 9 of these predicted inhibitions biochemically, and validated the novel inhibition of kallikrein 5 (KLK5) by serpin B12 (SERPINB12), previously an orphan inhibitor.

## EXPERIMENTAL PROCEDURES

### 

#### 

##### Proteases and Protease Inhibitor Data

Protease and protease inhibitor data and coexpression matrices used throughout the analyses are available for download at http://hdl.handle.net/11272/10472. Protease and inhibitor class, family, cleavage, and inhibitor information were extracted from the MEROPS database (http://merops.sanger.ac.uk/) (21) version 9.9 on September 30, 2013. MEROPS identifiers were used to classify proteases and inhibitors into classes and families as described on the MEROPS website.

##### Protein–Protein Interaction Networks

Protein-protein interaction (PPI) data from Human Integrated Protein-Protein Interaction Reference (24) version 1.5 were downloaded on June 12, 2013. PPI data from high-throughput experiments were downloaded from BioGRID (25) on October 11, 2013. PPI data from (26) were downloaded on October 11, 2013. Experiments with up to 100 identified PPIs were considered low throughput, those with 100–1,000 PPIs were labeled medium throughput, and those with more than 1,000 PPIs were deemed high throughput.

##### Protein Localization

Protein localization information was downloaded from three sources: LocDB (27) (data downloaded November 19, 2013), the Human Protein Atlas (28) (downloaded November 12, 2013.), and Gene Ontology (GO) annotation using the *hgu95av2.db* package in R (29) (downloaded August 8, 2013). For each dataset, annotations were mapped to GO terms and annotation trees for each protein were generated using the *GOstats* package in R (29). For LocDB, primary and secondary localization information was combined for each protein. Main and other localization data from the Human Protein Atlas were used if the reliability was annotated as *High*, *Medium*, or *Supportive*. GO annotations were retained if the evidence code was one of EXP, IDA, IPI, IGI, IMP, IEP, or TAS.

##### Coexpression Networks

Genome Tissue Expression Atlas (GTEx) data (30) were downloaded on January 31, 2013. Gene Expression Omnibus Series 7307 expression data were downloaded from the database Gemma (31) on June 26, 2013. Other microarray-based expression datasets used in meta-coexpression analysis were downloaded from Gemma on January 18, 2013 and are listed in supplemental Table S6. Gene correlation was calculated using the *cor* function in R (29). Partial correlation was calculated using the *ppcor* package in R. Full datasets or subsets were used as inputs as explained in the results section and in supplemental Table S5.

##### Phylogenetic Profiling

Phylogenetic profile data were constructed by downloading mappings from human proteins to other species from InParanoid (32). Mappings were binarized into 0 (absent) and 1 (present) for the binary networks before calculating the fraction of agreement (where the genes are absent or present in both organisms), Pearson correlation (*cor* package in R (29)) or mutual information (*entropy* package in R) for all pairs of genes. The Bits network was constructed by multiplying InParanoid scores with the bit score for each cluster and the calculating Pearson correlation (*cor* package in R).

##### Machine Learning

Machine learning algorithms were run using the *caret* package. 60% of pairs were used for training and 40% for testing. Parameters picked by cross-validation were mtry of 2 for random forest and C of 0.1 and sigma of 0.2 for the radial support vector machine.

##### Biochemical Validation Experiments

Coagulation factor 11 (F11), coagulation factor 12 (F12), plasma kallikrein (KLKB1), and the chromogenic substrates for F11 (2366 Catalogue# S821090), F12 (Catalogue# S820340), and for KLKB1 (S2302) were from DiaPharma. Chromogenic substrates were measured at an emission wavelength of 405 nm as recommended by the suppliers. KLK5 (Catalogue# 1108-S.E.-010) and its quenched fluorescent substrate (Catalogue# ES011) and kallikrein 5 (KLK7, Catalogue# 2624-S.E.-010) and its quenched fluorescent substrate (Catalogue# ES002) were from R&D Systems. Cleavage of quenched fluorescent substrates was measured using excitation/emission wavelengths of 380/460 nm for KLK5 and 320/405 nm for KLK7 as recommended by the suppliers. SERPINB12 was kindly provided by Dr. G. A. Silverman (Children's Hospital of Pittsburgh); serpin A4 was kindly provided by Dr. J. Chao (Medical University of South Carolina); murine serpin B8 was from Sino Biological, Inc. (Catalogue# 50215-M08B); and serpin B7 was from Creative BioMart (Catalogue# SERPINB7–2596H). Protease activity was measured after incubation for 1 h at 37 °C with and without serpins in a POLARstar OPTIMA plate reader (BMG Labtech). Substrate cleavage and protease inhibition assays were also analyzed by 10% SDS-PAGE and silver staining of proteins after incubation at a 1:1 ratio protease:inhibitor (w/w) for 1 h at 37 °C.

## RESULTS

Our results are organized around a presentation of our investigation of the predictive power of each of several data types we considered. We then describe a combined prediction approach that attempts to improve predictive power, biochemical validation of selected predictions, and finally a more in-depth investigation of coexpression patterns of proteases and inhibitors.

### 

#### 

##### PPIs

**Fig. 1.**
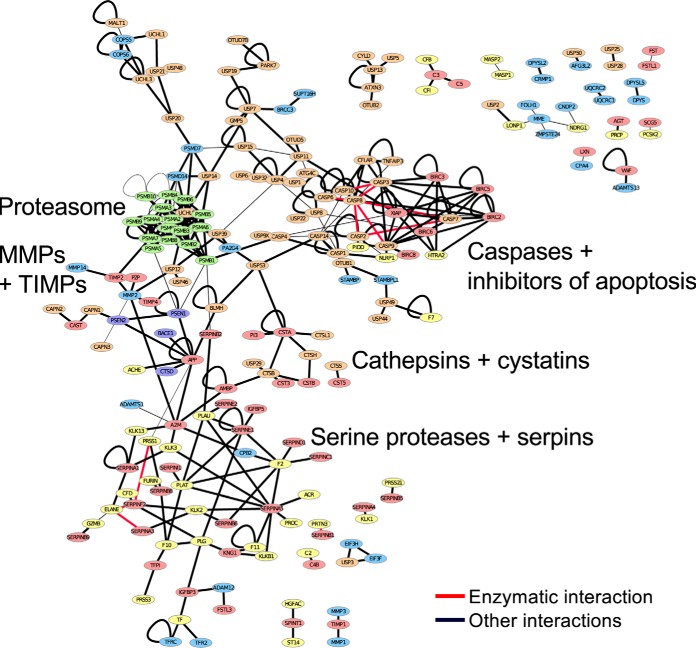
**Protein–protein interaction (PPI) networks of proteases and inhibitors.** PPI network of proteases and inhibitors based on the HIPPIE database with a HIPPIE score cutoff of 0.7. Nodes are colored according to their MEROPS class (proteases: green—threonine; blue—metallo; yellow—serine; orange—cysteine; purple—aspartic; inhibitors: red). Red edges are enzymatic interactions. Thickness of edges corresponds to the HIPPIE score of the interaction.

Our goal in considering general PPI data was to identify any relevant interactions that are not included in MEROPS. We analyzed PPI networks of proteases and their inhibitors from the databases HIPPIE (24) (supplemental Table S1) and BioGRID (25) (supplemental Table S2), and a literature-curated PPI network (26) (supplemental Table S3). Comparing 559 annotated cleavage and 325 inhibition interactions between proteases (including inactive proteases) and inhibitors from MEROPS to PPI data from HIPPIE (24), we found a PPI between protease and substrate for 187 known cleavages (33%) and between inhibitor and protease for 88 known inhibitions (27%). Figs. 1 and S1*A* show interactions annotated in HIPPIE between well-defined groups of proteases and inhibitors such as the proteasome, cathepsins, serum serine proteases, MMPs, and deubiquitin hydrolases. Confirming our earlier findings, (18) the connectivity in this network is heavily influenced by protease inhibitors (supplemental Fig. S1*B*).

We investigated the possibility that novel protease inhibitors of proteases might be hidden in the high-throughput PPI screens, which identify thousands of interactions but often without functional follow up. We collected 96 protease–inhibitor PPIs not already annotated as inhibitory interactions in MEROPS and examined all the original publications that served as references for these interactions in HIPPIE (supplemental Table S4). In 28 cases (29%), an inhibition of protease activity was observed in the original paper; in 20 (21%), an inhibition was inferred from complex formation in the source publication; and 14 (15%) interactions were based on a cleavage event of the inhibitor. Taken together, 62 (65%) of the 96 interactions were known protease web interactions that were simply not annotated in MEROPS, confirming the incomplete status of protease and protease inhibitor annotations reported previously (18). Of the remaining 34 interactions, 18 (19% of total) reflected a PPI not related to inhibition or cleavage, 3 (3%) were unclear interactions, and 13 (14%) PPIs were physical interactions between proteases and inhibitors with no mention of inhibitory activity and therefore potentially interesting novel inhibitions (Supplementary Results). Thus, PPI data not only reflect known protease interactions but also are potentially useful to predict novel inhibitory interactions. To identify additional inhibitory interactions, we further analyzed a BioGRID-derived (25) (supplemental Table S2, Fig. S1*C*) and a literature-curated PPI network (26) (supplemental Table S3, Fig. S1*D*). However, these networks were subject to study biases as described previously (33) and did not yield interesting predictions (Supplementary Results). These results motivated us to consider additional data types for inhibitory interaction prediction.

##### Coexpression Patterns

**Fig. 2.**
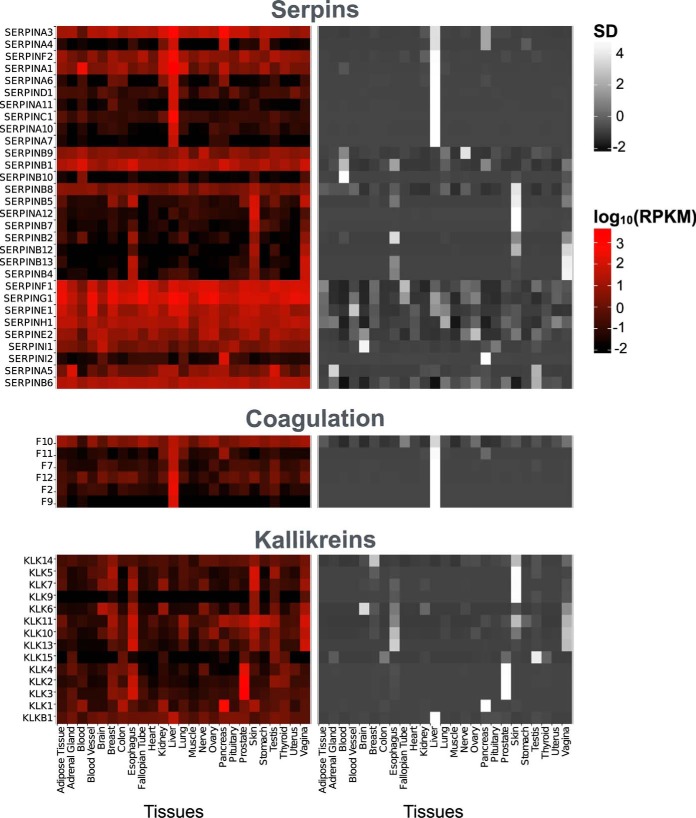
**Expression patterns.** Tissue RNA expression levels of groups of proteases and their inhibitors showing tissue-specific and broad expression patterns as well as correlation of expression patterns between serpins and serine proteases. Log_10_ transformed reads per kilobase of transcript per million mapped reads (log_10_(RPKM)) as obtained from GTEx (30) shown on the left. Zero values were set to 0.01 before log10 transformation. Normalized RPKMs for each gene are shown on the right and plotted as standard deviation from the mean (SD). Values were averaged across samples of each tissue.

We explored tissue expression profiles of proteases and inhibitors to seek useful patterns of coexpression, primarily in the GTEx (30) due to its high coverage (RNA-Seq data for 26 different tissues distributed over 1,660 samples). Expression patterns (Fig. 2) distinguish tissue-specific genes and housekeeping genes expressed in most or all tissues. For example, serpins either have a broad expression pattern across tissues (*e.g.* serpins E1, F1, and G1) or are specific to one or two tissues (*e.g.* serpins A1, C1, and D1), matching with known targets such as coagulation proteins and kallikreins (Fig. 2). Further examples are shown in supplemental Figs. S2 and S3.

We investigated the possibility of exploiting gene expression patterns to predict protease–inhibitor interactions. It has been observed in cell culture that expression of a protease inhibitor positively correlates with its target protease, which is suggested to counterbalance the cleavage potential of the protease (14, 15) or negatively correlates to facilitate proteolysis (16, 17). Such correlated expression (coexpression) is promising as a prediction tool because RNA expression is generally measured for all genes simultaneously, and it is thus less biased than *e.g.* protein interaction data (22). We calculated correlation values for all protease–inhibitor pairs (a coexpression matrix) and compared these to our gold standard of TP and TN inhibitory interactions derived from MEROPS.

We aimed to capture the variety of possible coexpression patterns observed (Fig. 2). Therefore, we generated 40 coexpression matrices based on different data and correlation methods to (summarized in supplemental Table S5). As demonstrated in supplemental Fig. S4, Pearson correlation (which is strongly influenced by samples with small values or zeros), captures tissue-specific expression patterns whereas Spearman correlation requires correlation across all tissues. We therefore generated coexpression matrices using both measures on the entire GTEx dataset (supplemental Fig. S5*A*). In addition, to simultaneously capture both patterns of coexpression, we generated a matrix using the maximum of Pearson and Spearman correlation coefficients for each pairs of proteins (GTEX_All_Max). We then generated coexpression matrices (Pearson, Spearman, and maximum of both) using partial correlation, which might help resolve complex correlation patterns between multiple variables (genes). To capture tissue-specific correlation, we generated coexpression matrices in subsets of GTEx limited to only one tissue and one matrix representing the average of the tissue-specific matrices (each both for Pearson and Spearman correlation). For comparison, we also measured Pearson and Spearman correlation coefficients in a large dataset based on mRNA microarrays (GEO-ID: GSE7307, 677 samples from over 100 tissues, supplemental Fig. S5*B*). Finally, with the aim of deriving a more robust coexpression result, we performed a meta-analysis (34, 35) of gene expression over multiple microarray datasets (listed in supplemental Table S6 and shown in Fig. S5*C*) across all tissues and in a tissue-specific manner in two ways: (i) datasets were merged into one large dataset (Merged) and (ii) Pearson correlation coefficients were obtained from each dataset and then averaged for each gene pair (Averaged (22)).

**Fig. 3.**
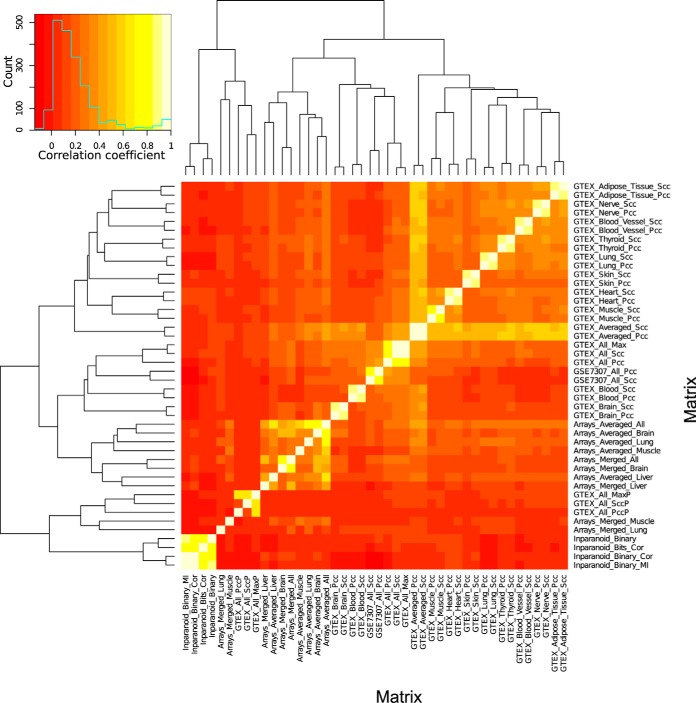
**Coexpression and phylogenetic similarity matrices.** Heatmap of correlations values (agreement) show similarity and dissimilarity of matrices described in Supplementary results and Table S5. For two matrices, the Pearson correlation coefficient across all coexpression values in both matrices is shown.

**Fig. 4.**
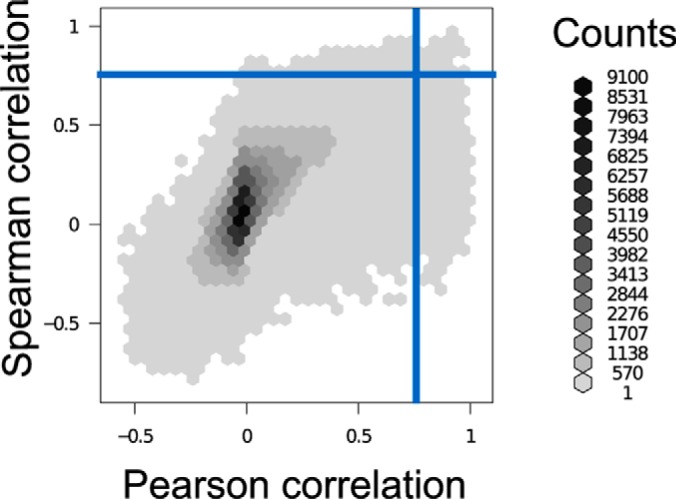
**Effect of threshold on predicted pairs in two correlated matrices.** Binned scatterplot of GTEX_All_Pcc (Pearson) and GTEX_All_Scc (Spearman) coexpression values for all pairs of proteins. Counts are number of pairs in each bin. Blue lines indicate a threshold of the mean plus two standard deviations. The overlap in predicted pairs above threshold (*top right corner*) is small despite the high correlation of these matrices.

The resulting coexpression matrices differed strongly in content depending on the methods and data used (Fig. 3, Supplementary Results). Coexpression values between some matrices were highly correlated overall (matrices GTEX_All_Pcc and GTEX_All_Scc with *r* = 0.67) as shown in Fig. 4, indicating similarity between methods. Yet, if predicting interacting pairs by applying a coexpression cutoff (blue lines in Fig. 4), these predictions resulted in a small overlap (*top right corner*). Predictive power thus needs to be assessed separately for each matrix.

**Fig. 5.**
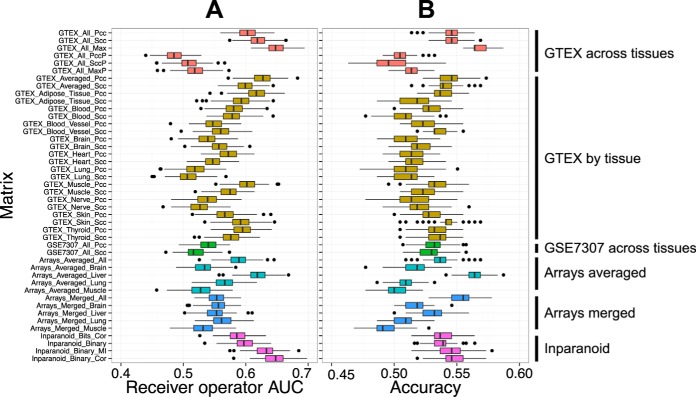
**Performance of coexpression and phylogenetic similarity matrices in predicting protease inhibition.** TPs are inhibitions (*n* = 218); TNs are specific inhibitor/protease pairs, where inhibition is enzymatically implausible. TNs were subsampled to reflect the number of TPs (*n* = 218, 200 times) to avoid effects resulting from an unbalanced gold standard. AUC values obtained from each sample are represented as boxplots. (*A*) AUC of the receiver/operator curve. (*B*) Accuracy of prediction (percentage of correct classifications), when predicting interactions as the top 10% of pairs of each matrix.

We compared the ability of coexpression matrices to predict protease inhibition (inhibitory protease–inhibitor pairs). We measured the area under the curve (AUC) of the receiver-operator characteristic for separating predefined TP pairs (annotated) from TN protease–inhibitor pairs (enzymatically implausible) using a given coexpression matrix. Fig. 5*A* shows that almost all matrices had some predictive value (better than random picking with AUC > 0.5). TP pairs thus had higher coexpression than TN pairs on average. However, considering the common perception that protease inhibitors are coexpressed with their target protease, this signal was surprisingly low (AUCs < 0.7). One explanation for this discrepancy might be that RNA levels do not correspond to protein levels, and thus proteins can be coexpressed whereas their mRNAs are not. We tested this possibility by creating a coexpression network based on proteomics quantification data in the Human Proteome Map (36), but this performed worse than the RNA networks (AUC of 0.6, data not shown). This poor performance might be due to noise in protein quantification or the small sample size of Human Proteome Map compared with GTEx. It thus remains unclear if protein data could be more informative than mRNA data.

Similar results were found when measuring accuracy of interaction prediction within the top 10% of coexpressed pairs of each matrix (Fig. 5*B*). Here, Array_Averaged_Liver and GTEX_All_Max methodologies had the highest signal. The strong correlation of Array_Averaged_Liver with protease web data was expected because many interactions are known between proteases and inhibitors that are expressed in liver as part of the well-understood complement and coagulation systems. However, this network is therefore biased to a subset of the protease web and performance would not generalize to other genes. The high AUC and accuracy of GTEX_All_Max demonstrated the usefulness this network, which combined tissue specific and across-tissue coexpression, making it the better candidate for prediction.

##### Phylogenetic Profiles

Phylogenetic similarity of two genes is a measure for their co-occurrence across a range of taxa and is reported to reflect functional relations (37). We created similarity matrices of phylogenetic profiles of proteases and inhibitors from the InParanoid (32) database using correlation of sequence similarity scores as well as correlation and mutual independence measures of binary presence/absence values of proteases across 162 species represented in the database (supplemental Table S5). The resulting matrices were very similar to each other (correlation 0.7–0.98), but dissimilar from the coexpression matrices (Fig. 3), indicating that this data source could be complementary to coexpression. Prediction performance of phylogenetic similarity matrices was generally comparable to coexpression matrices but weaker than GTEX_All_Max in the top pairs (Fig. 5*B*).

##### Colocalization

We next evaluated subcellular colocalization, which might enrich for interacting proteins. We obtained localization annotations LocDB (27), the Human Protein Atlas (28), and GO (38). We defined four groups of localization: (i) CO, colocalized for proteins sharing localization annotation; (ii) AT, antilocalized where one protein was extracellular and the other was in the cytosol or where one protein was in an organelle and the other in the cytosol (so that they meet upon cellular stimuli); (iii) NC, not colocalized where neither of the above was the case; and (iv) NA, not annotated, where subcellular localization was not annotated for one or both of the proteins. The use of colocalization enriched TP interactions considerably (Table I). However, colocalization also reduced the number of remaining pairs (the search space) significantly, mostly because of lack of annotation (NA). Using colocalization for novel predictions might increase specificity but substantially reduce sensitivity.

**Table I:** TP, TN, and remaining inhibitor–protease pairs after applying colocalization filters

	TN	TP	TP:TN	Remaining pairs
All	6,990	294	4.21%	32,368
Colocalized	430	65	15.12%	1,747
Antilocalized	466	30	6.44%	1,757
Information missing	5,338	159	2.98%	25,135
Remaining	756	40	5.29%	3,729

##### Coannotation and Comentioning in the Literature

Interacting genes often participate in similar cellular functions, and so it is possible to predict gene interactions based on their annotation patterns (39). We considered coannotation and comention as predictive features but dismissed both on theoretical and practical grounds. As we observed above for PPI data, utilizing this approach it is hard to distinguish between results that are *de novo* predictions, where novel interactions or functions are predicted, and mere retrieval of information already present in the literature but not yet annotated to databases (13). A related serious difficulty with this approach is that annotation is strongly biased by patterns of publication and gene annotation (40). Furthermore, if there were GO annotations linking a protease–inhibitor pair or in the literature, the interaction would likely already have been characterized biochemically. Estimation of prediction performance based on coannotation would thus appear overoptimistic. Predicted pairs would represent examples of information retrieval and not *de novo* predicted pairs. Because of the bias in annotation, poorly characterized proteins would likely never be predicted to be associated. Furthermore, many proteases and inhibitors are functionally related in cascades or biological processes, without having physical interactions, while here we were only interested in direct interactions, especially between previously unconnected cascades. Overall, comention and coannotation lack the coverage and detail required to make predictions about particular novel candidate pairs, and so we disregarded this feature.

##### Predicting Novel Inhibitions

In the face of poor prediction performance of the individual input data types assayed above, we hypothesized that a combination of matrices would improve prediction. However, we did not observe improvement when combining the different prediction matrices in machine learning classifiers (Supplementary Results, Fig. S6). Therefore, we focused on the best performing individual matrix for prediction, GTEX_Max, selecting a coexpression threshold of 0.6. This threshold enriching TP 5.5-fold in comparison to TN as shown by TP to TN ratio (TP:TN, Table II). We attempted to combine coexpression with colocalization. Colocalization of protease–inhibitor pairs further enriched TPs threefold and antilocalization enriched results 2.4-fold.

**Table II:** TP, TN, and remaining inhibitor–protease pairs after applying colocalization and coexpression filters

	TP	TN	TP:TN	Remaining pairs
Total	294	6,990	1:24	32,368
Coexpressed (R > 0.6)	46	205	1:4.5	1,237
Coexpressed and colocalized	10	18	1:1.8	112
Coexpressed and antilocalized	6	14	1:2.3	73

Nonetheless, colocalization information was missing for many proteins, thus limiting and biasing predictions. We therefore also included pairs where no localization information was available for one of the proteins. Finally, we removed all enzymatically implausible pairs, retaining only those where (i) it is known that the inhibitor blocks a protease from the same family as the predicted target protease or (ii) it is known that inhibition of the protease occurs by an inhibitor from the same family as the predicted cognate inhibitor. These two filters reduced the search space from 1,239 coexpressed pairs to 270 pairs. We anticipated that the incorporation of enzymatic constraint would greatly increase the precision of predictions. A loss of sensitivity is possible if all target protease families of an inhibitor or all inhibitor family members of a target protease are not annotated as such, but we considered this unlikely since relevant inhibitor families are known for most proteases. Inhibitor–protease pairs meeting these criteria are shown in supplemental Fig. S7 and listed in supplemental Table S7.

To evaluate the predicted protease–inhibitor pairs, we selected a number of pairs for biochemical experiments: inhibition of factor XI (F11), factor XII (F12), and KLKB1 by kallistatin (SERPINA4) (three pairs) as well as inhibition of KLK5 and kallikrein 7 (KLK7) by serpins B7, B8, and B12 (six pairs). It is important to note that we evaluated predictions without biases resulting from selecting the most likely pairs. Instead, we selected pairs of biologically interest and availability of reagents. GTEx showed high expression of kallistatin in liver together with the coagulation proteases factor XI, factor XII, and plasma kallikrein. Whereas this indicated an interesting new role for kallistatin, the predictions were indeed risky since all of the four proteins are well studied and newly discovered inhibition is therefore unlikely. Moreover, liver is known to express and secrete many serum proteases and inhibitors, and so there was additional likelihood that these predicted pairs would not be true. On the other hand, serpins B7, B8, and B12 are little studied inhibitors and thus biologically interesting interaction partners of KLK5 and KLK7.

**Fig. 6.**
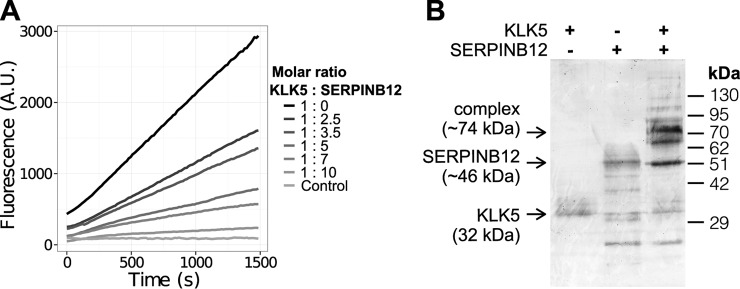
**Inhibition of KLK5 by SERPINB12 (*A*) Cleavage of the quenched fluorescent substrate ES011 by KLK5 was followed over time after preincubation with different mole ratios of SERPINB12 (as indicated).** A decrease in KLK5 activity (A.U.—arbitrary units) with increasing SERPINB12 confirms that SERPINB12 inhibits KLK5 as predicted. (*B*) Silver-stained 10% SDS-PAGE gel of KLK5, SERPINB12, and the inhibitory KLK5:SERPINB12 complex, indicating that the serpin is covalently bound to the protease. Serpins form metastable kinetically trapped folding states that are crucial for the inhibitory mechanism. Thus, serpins can occur in multiple folded states, some of which expose the reactive center loop as bait for protease cleavage. The presence of other folded states also indicates that N or C-terminal loops and strands may be cleaved by KLK5 depending on the conformations of the serpin present, which may shield or expose other potential cleavage sites. Thus, the lower molecular weight forms of the serpin–KLK5 covalent complex likely represent cleavage of N or C peptides of the serpin in addition to the inhibitory cleavage in the reactive center loop. Further autocleavage of KLK5 results in lower molecular weight forms of a still active protease, which may also form lower molecular weight inhibitory complexes.

We tested all pairs by fluorescent substrate cleavage assays *in vitro*. We found no new targets for kallistatin among the three serum proteases but did observe inhibition of KLK5 by SERPINB12 (Fig. 6*A*), which we confirmed by analysis of covalent complex formation on SDS-polyacrylamide gels (Fig. 6*B*). Given that these proteins are coexpressed and therefore found in the same tissue, we conclude that this interaction is also likely to be physiologically relevant. The interaction could also represent an interesting drug target since KLK5 is a major regulator in a number of diseases (41).

##### Limited Coexpression of Proteases and Their Inhibitors

**Fig. 7.**
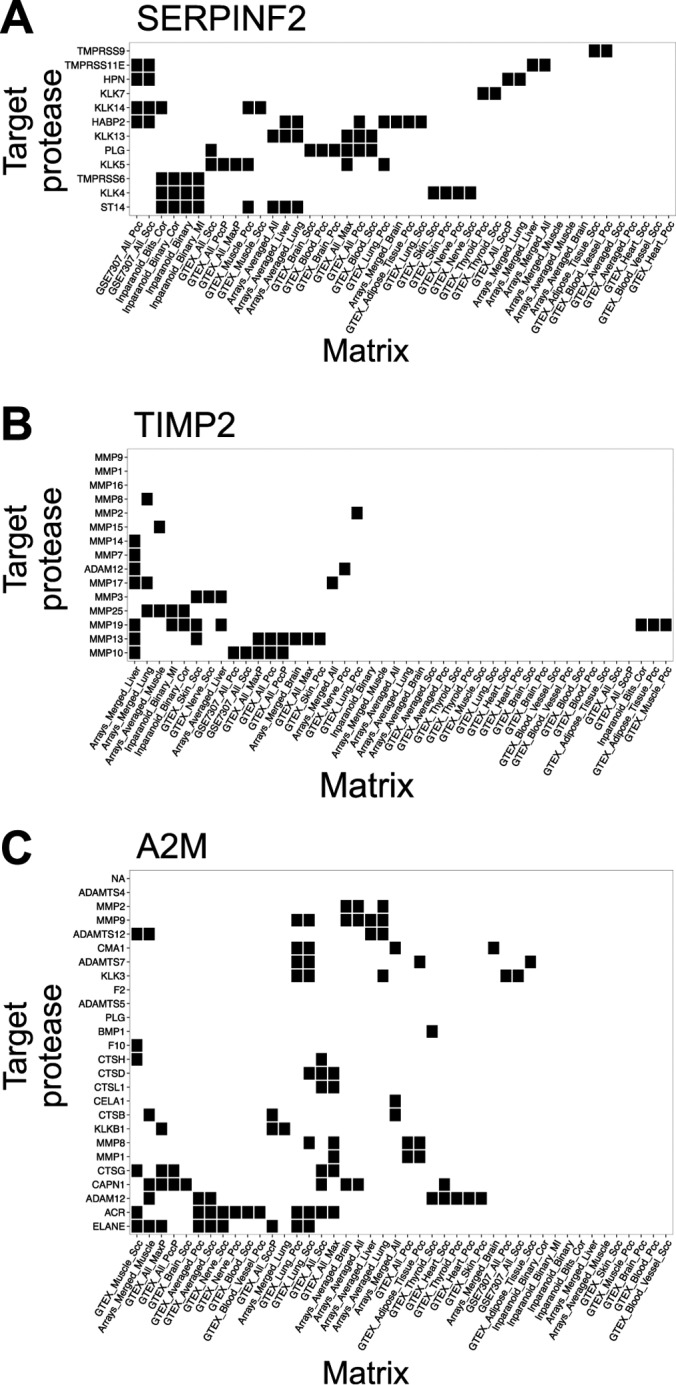
**Recovery of known inhibitor–protease pairs in matrices.** Recovered pairs among the top 10% of ranked pairs in each matrix marked in black. Columns are coexpression and phylogenetic similarity matrices from supplemental Table S5. Rows are genes of proteases that are annotated to be inhibited by (*A*) SERPINF2 (alpha-2-antiplasmin), (*B*) TIMP2 (tissue inhibitor of metalloproteinases), and (*C*) A2M (alpha 2 macroglobulin).

We were intrigued by the low coexpression of protease–inhibitor pairs (Fig. 5) and the limited success in validating predicted coexpression pairs. To analyze differences in predictions of our prediction matrices, we compared the true inhibitions that are retrieved when collecting only the most coexpressed protease–inhibitor pairs (top 10%) from each matrix. This analysis confirmed that GTEX_All_Max captured most pairs predicted by other coexpression matrices and was thus the better choice for prediction (supplemental Fig. S8, Supplementary Results). We also tested whether a more comprehensive dataset would improve predictions by basing our predictions using updated versions of GTEx (version 4 instead of 3 with 2,921 instead of 1,660 samples) and MEROPS (version 11.0) but did not see any improvement (supplemental Figs. S9 and S10). To further understand the low correlation between coexpression and the protease inhibition, we investigated coexpression of exemplary, well-studied inhibitor–protease pairs. First, we examined serpins that regulate coagulation (*e.g.* antithrombin III (SERPINC1) and alpha-2-antiplasmin (SERPINF2)). Indeed, these serpins were coexpressed with coagulation proteases in liver, which, as noted above, is the case for many serum proteins destined for secretion to the circulation. However, these serpins are annotated to inhibit many additional proteases. For example, SERPINF2 inhibits KLK 4, 5, 7, 13, and 14 that are not highly expressed in liver, have uncorrelated expression patterns from SERPINF2 (Fig. 2) and are thus not retrieved by coexpression analysis (Fig. 7*A*). Yet, these interactions are biochemically meaningful, as serpins are exported from the liver to the serum and thus transported through the body, where they encounter and inhibit proteases with uncorrelated expression patterns. Thus, as we showed before, coexpression in exocrine tissues is limited in predicting such protein interactions.

These observations were not limited to serpins but applied to all groups of inhibitors: For example, TIMP 2 (Fig. 7*B*) was not coexpressed with any of its annotated target proteases with a few exceptions (MMP2 or MMP14), which were drowned out by the number of other annotated targets. In the extreme case, alpha-2-macroglobulin (Fig. 7*C*), a highly multifunctional protease inhibitor that inhibits multiple classes of proteases, is also only coexpressed with a small portion of proteases. However, alpha-2-macroglobulin is present in blood plasma and so can reach most tissues (especially in inflammation were blood vessel permeability is increased) (23) and inhibit extracellular proteases or intracellular proteases that are secreted (*e.g.* by neutrophils (*e.g.* serine proteases and MMPs)) or released from damaged cells. These examples show that expression patterns of inhibitors are often correlated with the expression pattern of some (possibly the most relevant) targets but uncorrelated with additional biologically relevant targets.

##### Protease–Inhibitor Predictions Comparison to General Protein Interaction Predictions

**Fig. 8.**
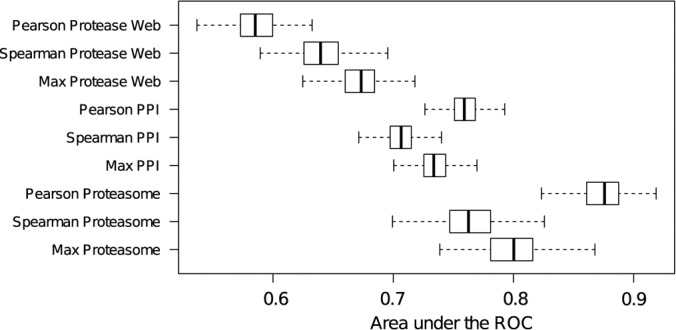
**Prediction of PPI and predictions of protease inhibition.** Area under the receiver-operator curve for predictions of protease inhibitions (Protease Web), protein–protein interactions from HIPPIE (PPI, Fig. 1), and interactions of proteasome components in HIPPIE (Proteasome, Fig. 1). Predictions are based on the coexpression matrices GTEX_All_Pcc (Pearson), GTEX_All_Scc (Spearman), and GTEX_All_Max (Max).

We compared the performance of our coexpression matrices in predicting protease–inhibitor interactions to the performance achieved when predicting general protein interactions in the HIPPIE network (Fig. 1), using interactions annotated in HIPPIE as TPs and random interactions as TNs. Prediction of protein interactions showed higher accuracy than predictions of protease inhibitions (Fig. 8). This difference was even stronger when focusing on proteasomal protein complex, demonstrating that prediction of protease inhibitions is indeed a difficult task compared with the prediction of general protein interactions.

To assess whether predictions of generic protein interactions in state-of-the-art methodology are useful in predicting protease inhibitions, we obtained predicted interaction partners of protease inhibitors from PrePPI (42). Without our enzymatic constraints, these predictions included many predictions that were irrelevant for the identification of protease inhibitors. For example, predicted partners of kallistatin (SERPINA4) in PrePPI included many proteins that were not proteases or were themselves protease inhibitors (*e.g.* SERPIND1 and SERPINC1). Focusing on interactions between inhibitors and proteases (predicted probability of 0.9 or higher), we identified 241 predictions. 41 interactions overlapped with our 270 predictions despite the use of different methodology and underlying data. It is not possible to determine the accuracy of PrePPI predictions since PrePPI uses structural constraints and GO annotations, which partially correspond to the annotations we used to build the gold standard. However, despite these filters, 7 of the 51 interactions of serpins included metalloproteases, and none of the eight predicted interactions of cystatins included cysteine proteases, indicating that these predictions lack specificity and are not easily applicable to the prediction of inhibitory protease interactions.

## DISCUSSION

We observed that coexpression was very limited in utility in predicting protease–inhibitor pairs, contrary to previous findings suggesting proteases and their inhibitors have correlated expression (14 and 15). Our results suggest this is not a general principle, at least at the RNA level. We did not observe specific coexpression of many protease inhibitors with their target proteases despite our extensive efforts in calculating coexpression across human tissues and within tissues, based on proteomics and using machine learning to combine networks. As demonstrated by our analysis (Fig. 7), we deciphered a biological reason for the delinking of protease–inhibitor coexpression in the mobility of proteins in an open biological system with interactions between tissues and cells. While coexpression might thus be better observed at the protein level, we also did not observe this, possibly due to the limited quality and extent of protein quantification. We further hypothesize that in complex networks, where an inhibitor inhibits many proteases and *vice versa*, the expression pattern of one gene represents a combination of the patterns of its interactors and is thus not clearly correlated with any one of them individually. However, our attempt to address this by using partial correlations did not provide support for this notion.

In addition to many true inhibitor–protease pairs not coexpressing, we also found that coexpressed pairs are often not inhibitory. We hypothesize that this is due to pathways of genes, where a gene can be coexpressed with other genes in the same pathway that are not direct interactors. In our data, one example is the coexpression of SERPINB12 with KLK5 and KLK7. SERPINB12 inhibits KLK5 but not KLK7. KLK5 is also an activator of KLK7 (41). Whereas SERPINB12 and KLK7 are involved in the same pathway and coexpressed, they do not interact directly. Nonetheless, inhibition of KLK5 by SERPINB12 prevents activation of KLK7 and thus indirectly inhibits KLK7. Thus, network effects (18) might explain some false positives in protein interaction prediction studies.

Due to the complexity of biological networks, identification of biochemically relevant protein interactions remains an intricate problem. Prediction of inhibitors of proteases provided in-depth insights into the related difficulties since it allows a cleaner definition of TP and TN examples than general protein interaction prediction. We emphasize that biochemical evaluations are useful to give realistic estimates of expected performance because computational evaluation such as cross-validation performance is a very poor predictor of how a guilt-by-association method will do in reality (43). Our performance results are in line with many previous evaluations of guilt-by-association methods (43), and we conclude that much higher validation rates are unrealistic in unbiased biochemical experiments. Indeed, no prediction method has been adopted by biochemists for routine prioritization (13), despite reports of improved prediction performance (9, 42). The best performing biochemically validated protein interaction prediction method (PrePPI) (9, 42) was reported to be successful in 15 of 19 experiments (suggesting ∼79% precision). However, in that case, predictions were guided by GO annotation, possibly reflecting information retrieval rather than the harder *de novo* predictions, and were hand selected for validation based on plausibility, which can significantly bias performance evaluation. Therefore, the reported performance for PrePPI is likely overoptimistic. Indeed, in a recent mass spectrometry screen of interaction partners of adenosine monophosphate-activated protein kinase-α1 and -β1 (44), only 63 of the 381 biochemically identified interaction partners overlapped with the 1,235 interactors predicted by PrePPI, giving only ∼5% precision. We posit that low validation rates in realistic settings can explain the limited use of prediction tools in guiding biochemical experiments.

We also revealed strengths and weaknesses of individual input features in our focused analysis. One important input feature was the prior knowledge of structural constraints, namely the knowledge of protease and inhibitor classes. We hypothesized that this feature would greatly simplify the inhibitor prediction task by narrowing and focusing the search space compared with the general protein interaction prediction problem. Without enzymatic constraints, common protein–protein interaction predictions included many irrelevant predictions, as we demonstrated in the case of PrePPI. In our analysis, protease-specific structural features significantly reduced the search space to relevant interactions but also did not discern specificity between closely related protein family members. We conclude that such structural information as well as the related sequence-based input used in phylogenetic similarity decreases the search space to plausible pairs but is not specific enough to identify individual interactions.

Overall, we conclude that computational interaction prediction remains challenging and with the current state of data, and methods seem unable to accomplish the task with sufficient specificity to reliably replace biochemical experiments. Improvements may come from more comprehensive expression studies and proteomics quantification as well as a better definition and larger numbers of TP and TN examples used for training to identify pertinent patterns. This will improve as more data are uploaded to community databases such as MEROPS and TopFIND for proteases and inhibitors. We caution against overoptimistic estimates of performance based on aggregating diverse data in a black box algorithm and overtrusting cross-validations based on flawed gold standards (43, 45). Equally problematic are biases toward well-studied proteins (40) by relying on functional or localization data that can lead to valid but less interesting interactions. By carefully selecting and combining features in a transparent method that is more analogous to a biologist's reasoning, we can clarify limitations of features and thus should build confidence among the users of predictions.

## DATA AVAILABILITY

Protease and protease inhibitor data and coexpression matrices used throughout the analyses are available for download at http://hdl.handle.net/11272/10472.

## Supplementary Material

Supplemental Data

## Footnotes

^1^ The abbreviations used are:

TP: true positives
TN: true negatives
PPI: protein-protein interaction
MMP: matrix metalloproteinase
AUC: area under the curve
ROC: receiver operating characteristic
RPKM: reads per kilobase of transcript per million mapped reads
GO: Gene Ontology
EXP: Inferred from Experiment
IDA: Inferred from Direct Assay
IPI: Inferred from Physical Interaction
IGI: Inferred from Genetic Interaction
IMP: Inferred from Mutant Phenotype
IEP: Inferred from Expression Pattern
TAS: Traceable Author Statement.
