# Supplementary material for: Protease-Inhibitor Interaction Predictions: Lessons on the Complexity of Protein–Protein Interactions
Source: Mol Cell Proteomics. 2017 Apr 6;16(6):1038–51. doi: 10.1074/mcp.M116.065706 (PMC5461536; doi:10.1074/mcp.M116.065706)
Supplement: Supplemental Data [file supp_16_6_1038__index.html]

Protease-inhibitor interaction predictions: Lessons on the complexity of protein-protein interactions — Protease-Inhibitor Interaction Predictions: Lessons on the Complexity of Protein–Protein Interactions — Bioinformatics Prediction of Protease–Inhibitor Interactions — Supplemental Data 

# Protease-Inhibitor Interaction Predictions: Lessons on the Complexity of Protein–Protein Interactions

## Supplemental Data

- SupplementResults (.pdf, 10.9 MB) - Supplementary Results and Figures
- SuppTables (.xlsx, 220 KB) - Supplementary Tables
