# Supplementary material for: Protease-Inhibitor Interaction Predictions: Lessons on the Complexity of Protein–Protein Interactions
Source: Mol Cell Proteomics. 2017 Apr 6;16(6):1038–51. doi: 10.1074/mcp.M116.065706 (PMC5461536; doi:10.1074/mcp.M116.065706)
Supplement: Supplemental Data [file 10.1074_M116.065706_mcp.M116.065706-1.pdf]

SUPPLEMENTARY TABLES

Table S1: **Complete list of interactions in the HIPPIE PPI network used to generate Figure 1A.**

Table S2: **Complete list of interactions in the BioGRID PPI network from Figure 1C.**

Table S3: **Complete list of interactions in the literature curated PPI network from Figure 1D.**

Table S4: **Protein-protein interaction pairs of protease with inhibitor predicted as "inhibition" from HIPPIE.**

Table S5: **Table of matrices of coexpression and phylogenetic similarity used throughout the manuscript.**

Table S6: **Microarray datasets used to generate coexpression matrices across datasets.**

Table S7: **Inhibitor-protease pairs predicted from coexpression and enzymatic plausibility.**

SUPPLEMENTARY RESULTS

Predicted novel inhibitions in HIPPIE

Of the 13 interactions in HIPPIE that suggested possible new protease/inhibitor interactions, 8 occurred between the ubiquitin hydrolase (DUB) USP19 and apoptosis inhibitors XIAP, BIRC2, and BIRC3 (1), and also between the DUB USP11 and XIAP (2). These interactions between apoptosis inhibitors (which are often ubiquitin E3 ligases) and DUBs are well documented in the literature, but to our knowledge it has not been shown if DUBs are inhibited by the apoptosis inhibitors. Potentially inhibitory PPIs in HIPPIE were also found between cystatin A and USP53 (2) or UCHL5 (3). Cystatin A is an inhibitor of cysteine-cathepsins. As DUBs are cysteine proteases, DUBs are potential targets of intracellular cystatin A, or one of the other intracellular cystatins F, B, or 11. Similarly, serpin B12, which is only reported to inhibit thrombin and plasmin, interacted with UCHL5 (3). Cathepsin B was reported to bind the protein bikunin (AMBP) (4). Both genes are highly expressed in tumor tissue (5), further indicating a functional interaction. The matrix metalloproteinase (MMP) inhibitor TIMP2 interacted with the metalloprotease puromycin-sensitive aminopeptidase NPEPPS (6), but TIMPs have only been shown to inhibit endopeptidases to date. Another PPI in HIPPIE was found between the cysteine protease inhibitor BIRC2 and the serine protease ABHD17A (or Fam108A1) (7). A complex of BIRC2 and caspase 8 (8) likely represents a true, but untested inhibition since BIRCs are endogenous inhibitors of caspases.

Analysis of a BioGRID derived and a literature curated PPI network

In addition to the data from HIPPIE (9), we compiled a high-throughput network of PPI using the larger BioGRID database (10). Most of this network (Figure S1C) is derived from just a few publications. One source identified binding partners of amyloid protein precursor (APP), shown by the cluster of red edges around APP. Another source identified binding partners (blue edges) of DUBs. Finally, two different publications (purple) were centered on the proteasome. We concluded that this network was strongly biased to the baits used in high-throughput screens, which were not relevant to our goal of

identifying protease inhibitors. As a third source of PPI information, we collected a literature curated PPI network (11) where we separated source papers by the number of interactions identified in each. The resulting network (Figure S1D) showed that most edges come from low throughput screens (grey). Medium throughput screens identified exclusively proteasome interactions. Finally, we observed that high throughput interactions between proteases and inhibitors are mostly also identified by low throughput methods and thus have likely been studied in detail and do not represent novel inhibition predictions.

Comparison of coexpression matrices

Pearson and Spearman correlation resulted in highly similar coexpression matrices, as seen in all matrices based on GTEX (12) data, across (r = 0.67) or within tissues (r > 0.8). Tissue-based coexpression yielded similar results, as seen in the GTEX tissue-based networks. In a few cases similarities were explained by tissue-similarity (r(GTEX_Heart_Pcc, GTEX_Muscle_Pcc) = 0.4), but in many cases similarities were likely due to constitutive coexpression of genes (similarities of adipose tissue, nerve, blood vessel, thyroid, and lung based coexpression matrices). Averaging tissue specific matrices seemed to have extracted generally coexpressed pairs, so that the GTEX_Averaged networks correlated with all tissue-based GTEX matrices (average r = 0.55). Interestingly, these in-tissue averaged coexpression (GTEX_Averaged) also correlated somewhat (r > 0.4) with across-tissue coexpression (GTEX_All), despite the methodological differences. Finally, coexpression matrices derived from using partial correlation showed similarities between partial Pearson and Spearman correlation, but differed from all other matrices. In conclusion, differences were large if data is used tissue-specific or across-tissues and averaging tissue-based networks extracts common features of tissue-specific networks. The effect of data sources was limited, as shown by the similarity of RNA-Seq based GTEX and microarray based GSE7307 across-tissue matrices, which was high (average r = 0.43) considering that these matrices stemmed from different samples, tissues, and instrumentation. Finally, meta-analysis of microarray datasets resulted in matrices different from the above GTEX based matrices, probably due to differences in samples and methodology. However,

again, similar methods seemed to result in similar matrices, so that Arrays_Averaged_All correlated with GTEX_Averaged (r = 0.46) and with the individual GTEX tissue-based matrices, and Array_Merged_All correlated somewhat with GTEX_Full_Pcc (r = 0.37), which was also most similar methodologically.

Combining features for prediction by machine learning

Having observed moderate correlation between the protease web and individual gene expression and phylogenetic similarity matrices, we considered combining these matrices as features to predict protease inhibition. We trained three common machine-learning algorithms to learn a function that combines the different matrices as features (the coexpression and phylogenetic similarity matrices from Table S5). We used random forest, linear discriminant analysis, and support vector machine to classify true positive and true negative inhibitor-protease pairs. Each pair had a feature vector containing one value for each matrix. We trained the classifiers on a training set of true positive and true negative interactions and compared their performance on a test set (Figure S6), where we also evaluated performance of the GTEX_All_Max matrix alone. While the AUCs of these classifiers were slightly above the AUC of GTEX_All_Max, the difference was small and, more importantly, the performance in the critical region of high sensitivity and high specificity (the lower-left hand part of the ROC curve) was indistinguishable. The low performance was probably either due the small number of positive training examples and/or the absence of learnable patterns in the data. Being unable to improve predictions from aggregation, we only used the GTEX_All_Max matrix for predicting true protease-inhibitor pairs. This matrix was the best performing among those tested, and furthermore, represents a measure of similarity that is transparent (being easily interpretable and visualized) and reasonable (fitting to our observation of expression patterns).

Comparison of top predictions

Investigating the overlap between the top predictions (10% of pairs) from each matrix (Figure S8A), we observed drastically different results between phylogenetic similarity and coexpression derived

matrices (Figure S8B) as previously observed in Figure 3 for the entire matrices. This analysis also confirmed GTEX_All_Max as the most reasonable choice for prediction, since it captures both types of tissue-specific and tissue-spanning coexpression patterns (Figure S8C) and most pairs identified in other coexpression matrices (Figure S8D).

SUPPLEMENTARY FIGURES

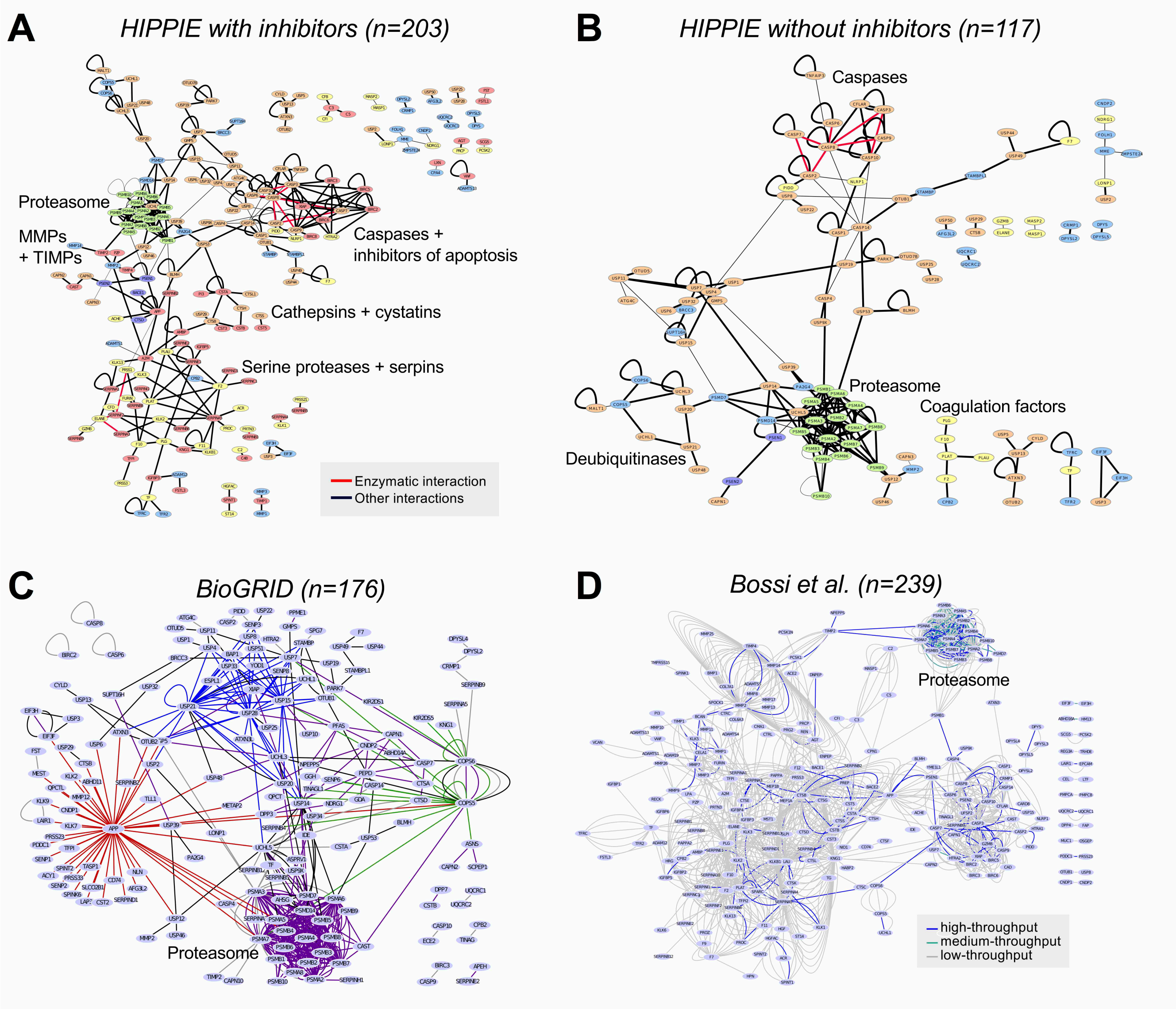

Figure S1. Protein-Protein Interaction (PPI) Networks Constructed for Proteases and Inhibitors

(A) and (B) PPI network based on the HIPPIE database with a HIPPIE score cutoff of 0.7. Isolated nodes were removed. Nodes are colored according to their MEROPS class (Proteases: green – threonine; blue – metallo; yellow – serine; orange – cysteine; purple – aspartic; Inhibitors: red). Red edges are enzymatic interactions, thickness of edges corresponds to the HIPPIE score of the interaction. (A) Interactions between proteases and inhibitors. (B) The same network as (A) without

inhibitors. Upon removal of inhibitors from the network, only known complexes such as the proteasome, DUBs and some blood coagulation proteases remained connected. (C) Network of interactions between proteases and inhibitors generated by high-throughput experiments from BioGRID. Edges are colored according to the study the edges were derived from (black - PMID19615732, green – PMID21145461, red – PMID21832049, blue – PMID22626734, purple – PMID22863883 and PMID22939629, grey – others, see Table S2). (D) Published network which was curated from current literature (11). Nodes are connected by multiple edges if the interaction was curated from multiple publications. Edges are colored to reflect the type of experiment (grey – low throughput, green – medium, blue – high throughput).

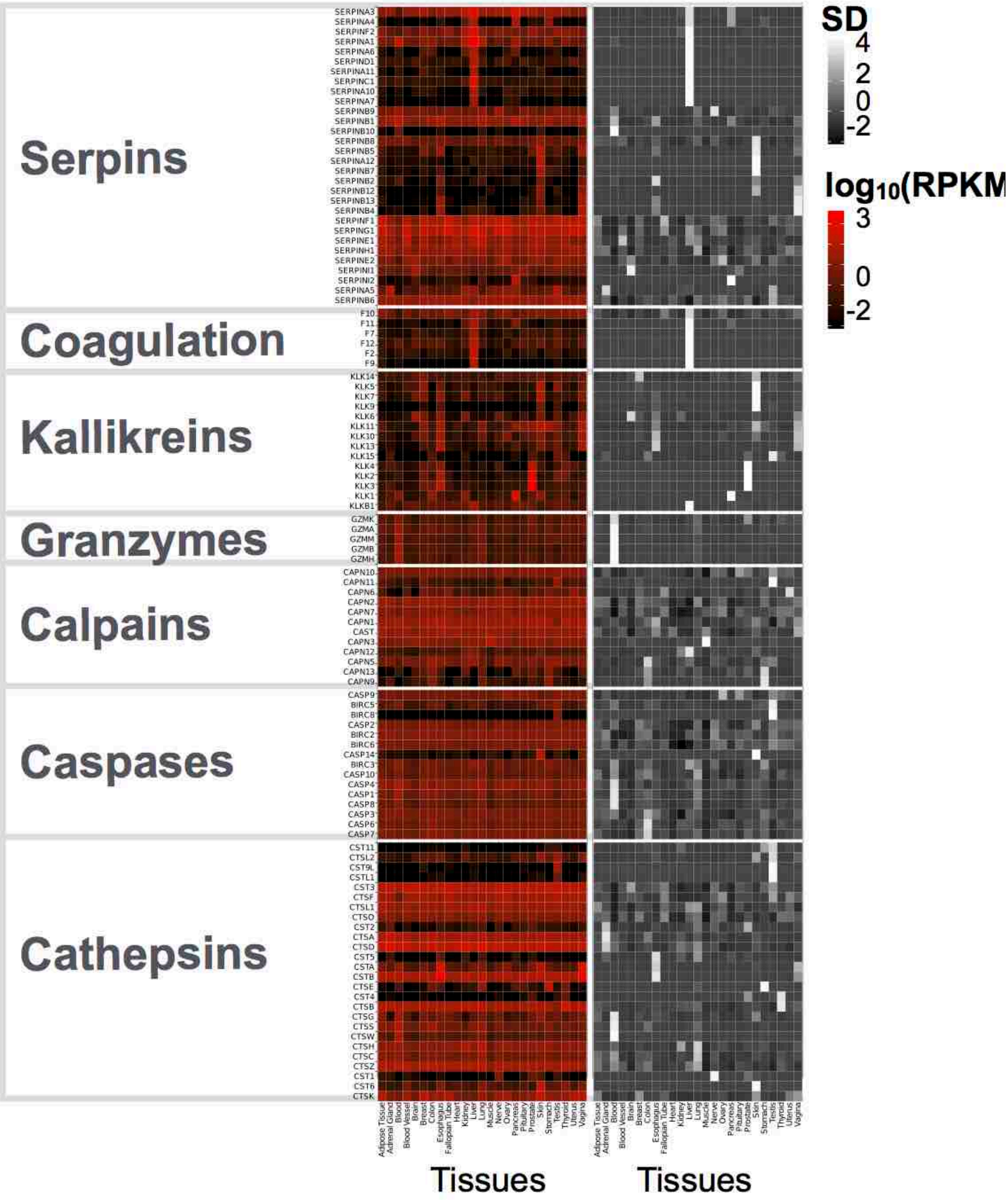

Serpins
Coagulation
Kallikreins
Granzymes
Calpains
Caspases
Cathepsins
SERPINA3
SERPINA4
SERPINF2
SERPINA1
SERPINA6
SERPIND1
SERPINA11
SERPINC1
SERPINA10
SERPINA7
SERPINB9
SERPINB1
SERPINB10
SERPINB8
SERPINB5
SERPINA12
SERPINB7
SERPINB2
SERPINB12
SERPINB13
SERPINB4
SERPINF1
SERPING1
SERPINE1
SERPINH1
SERPINE2
SERPINI1
SERPINI2
SERPINA5
SERPINB6
F10
F11
F7
F12
F2
F9
KLK14
KLK5
KLK7
KLK9
KLK6
KLK11
KLK10
KLK13
KLK15
KLK4
KLK2
KLK3
KLK1
KLKB1
GZMK
GZMA
GZMM
GZMB
GZMH
CAPN10
CAPN11
CAPN6
CAPN2
CAPN7
CAPN1
CAST
CAPN3
CAPN12
CAPN5
CAPN13
CAPN9
CASP9
BIRC5
BIRC8
CASP2
BIRC2
BIRC6
CASP14
BIRC3
CASP10
CASP4
CASP1
CASP8
CASP3
CASP6
CASP7
CST11
CTSL2
CST9L
CSTL1
CST3
CTSF
CTSL1
CTSO
CST2
CTSA
CTSD
CST5
CSTA
CSTB
CTSE
CST4
CTSB
CTSG
CTSS
CTSW
CTSH
CTSC
CTSZ
CST1
CST6
CTSK
Adipose Tissue
Adrenal Gland
Blood
Blood Vessel
Brain
Breast
Colon
Esophagus
Fallopian Tube
Heart
Kidney
Liver
Lung
Muscle
Nerve
Ovary
Pancreas
Pituitary
Prostate
Skin
Stomach
Testis
Thyroid
Uterus
Vagina
Tissues
Tissues
SD
4
2
0
-2
log10(RPKM
3
0
-2

Figure S2. Expression Patterns of Serine and Cysteine Proteases and their Inhibitors

Tissue RNA expression levels of groups of proteases and their inhibitors showing tissue-specific and broad expression patterns. Log10 transformed Reads Per Kilobase of transcript per Million mapped reads ($\log_{10}$(RPKM)) as obtained from GTEX (12) shown on the left. Zero values were set to 0.01 before log10 transformation. Normalized RPKMs for each gene are shown on the right and plotted as standard deviation from the mean (SD). Values were averaged across samples of each tissue.

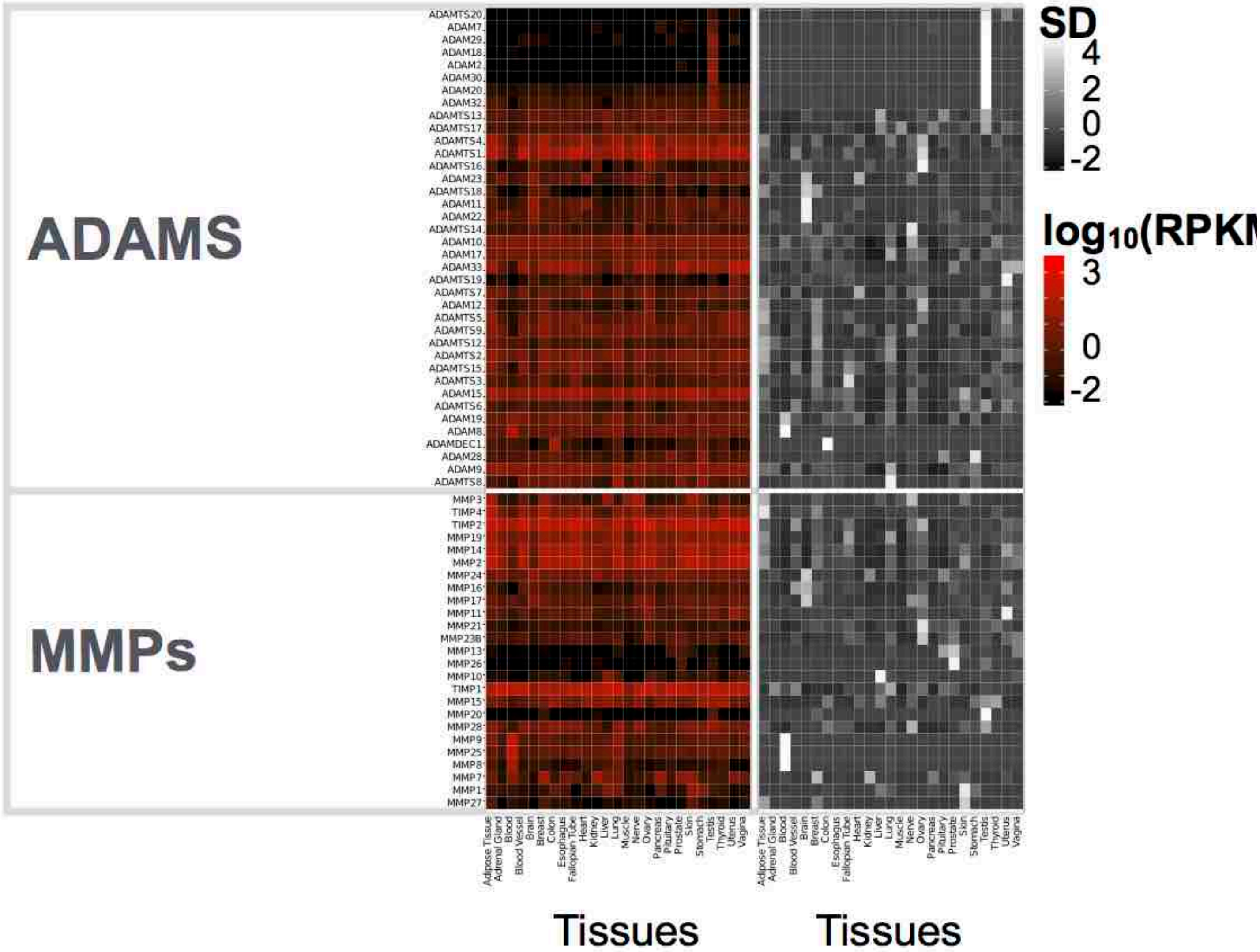

Figure S3. Expression Patterns of Metalloproteases and their Inhibitors

Tissue RNA expression levels of groups of proteases and their inhibitors showing tissue-specific and broad expression patterns. Log10 transformed Reads Per Kilobase of transcript per Million mapped reads ($\log_{10}$(RPKM)) as obtained from GTEX (12) shown on the left. Zero values were set to 0.01 before log10 transformation. Normalized RPKMs for each gene are shown on the right and plotted as standard deviation from the mean (SD). Values were averaged across samples of each tissue.

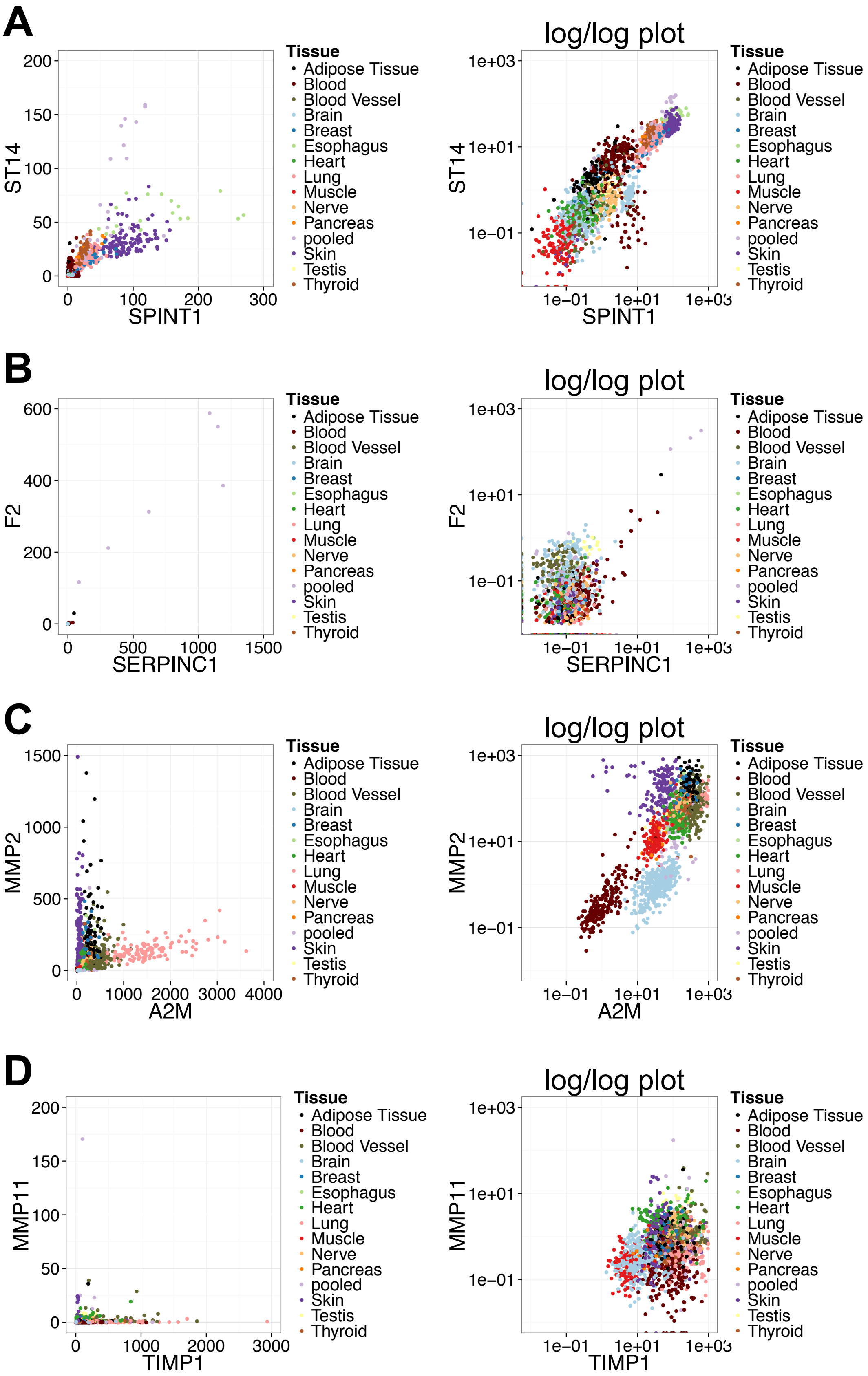
A
B
C
D
log/log plot
Tissue
Adipose Tissue
Blood
Blood Vessel
Brain
Breast
Esophagus
Heart
Lung
Muscle
Nerve
Pancreas
pooled
Skin
Testis
Thyroid
ST14
SPINT1
F2
SERPINC1
MMP2
A2M
MMP11
TIMP1
1e+03
1e+01
1e−01

Figure S4. Scatterplots of Expression for Selected Protease-Inhibitor Pairs

Normal scatter plot and log/log plot of (A) ST14/SPINT1, (B) F2/SERPINC1, (C) MMP2/A2M, and (D) MMP11/TIMP1. Pearson and Spearman correlation coefficients are both high when correlation throughout the range of expression levels is evident (A). Pearson correlation coefficients are high when both genes are expressed at high levels, even if they are not correlated in other tissues because it is sensitive to outliers (B). On the other hand, Spearman correlation coefficients are highest when the relation is maintained across most tissues and samples (C). Clearly both measures are low in cases where neither type of correlation exists (D).

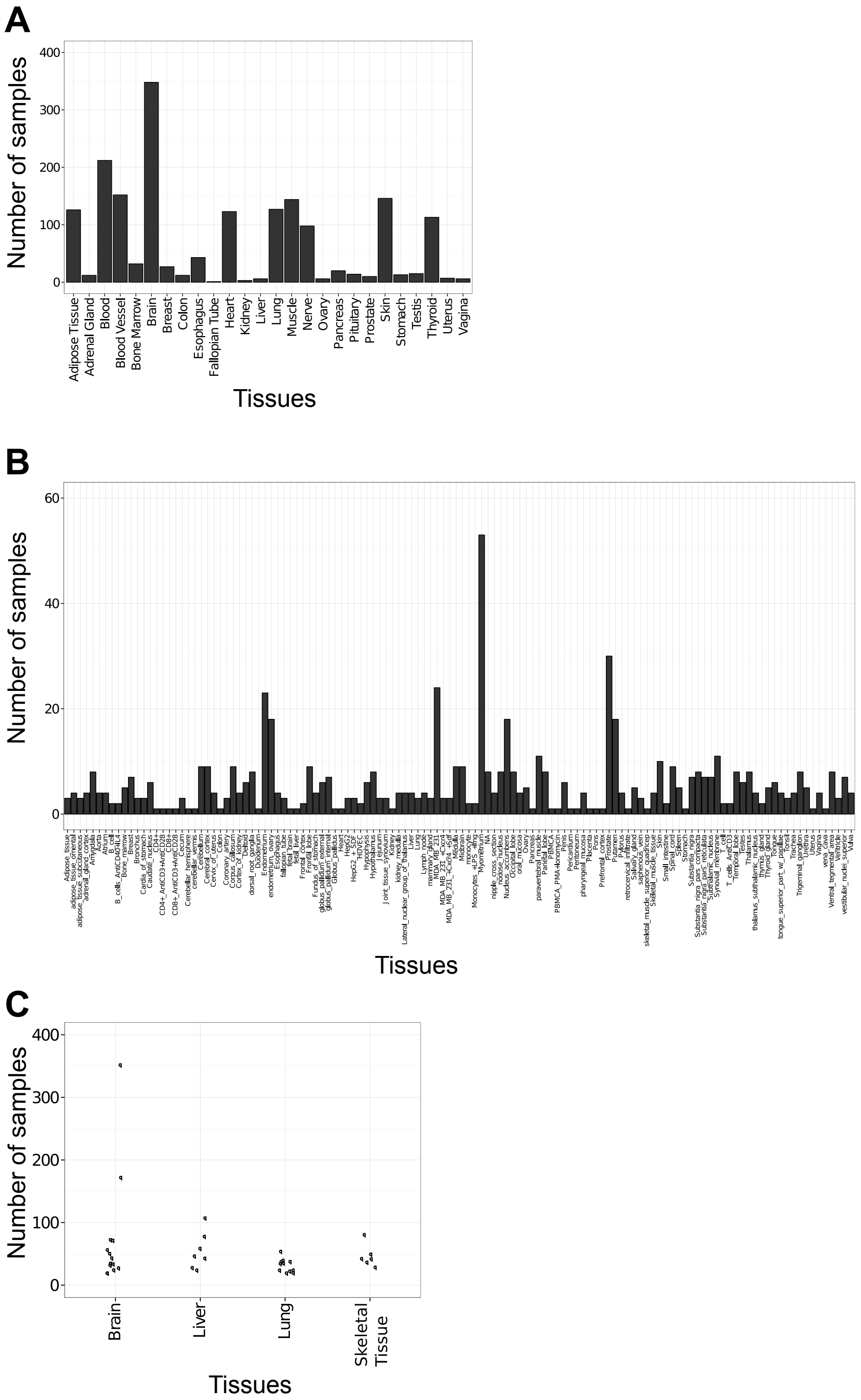

Figure S5. Tissue/Sample Distribution of Datasets

(A) Tissue distribution of samples in the GTEX dataset. (B) Tissue distribution of samples in the GSE7307 microarray dataset. (C) Number of samples in the microarray datasets used for coexpression meta-analysis.

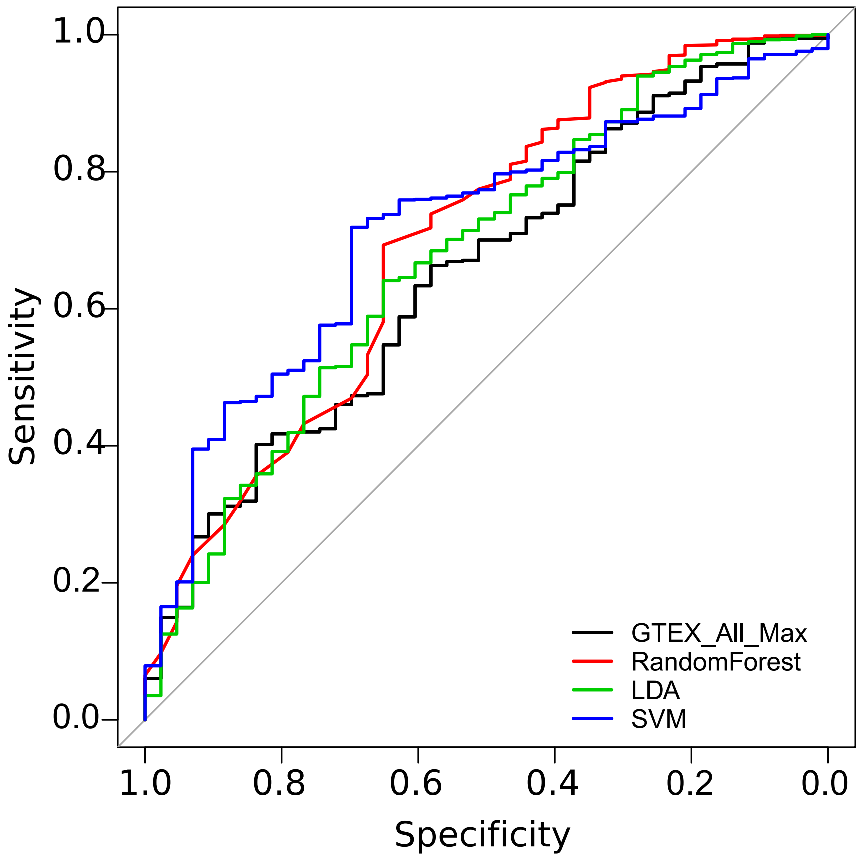

Figure S6. Performance of Machine Learning Algorithms in Predicting Protease-Inhibitor Pairs

Sensitivity plotted versus specificity for the three machine-learned classifiers random forest (red), linear discriminant analysis (green, LDA), and support vector machines (blue, SVM) compared to the coexpression matrix GTEX_All_Max (black). Performance is slightly higher overall (lines closer to the top left corner), but not in the top pairs (bottom left corner) comparing machine-learned classifiers to the original coexpression matrix GTEX_All_Max.

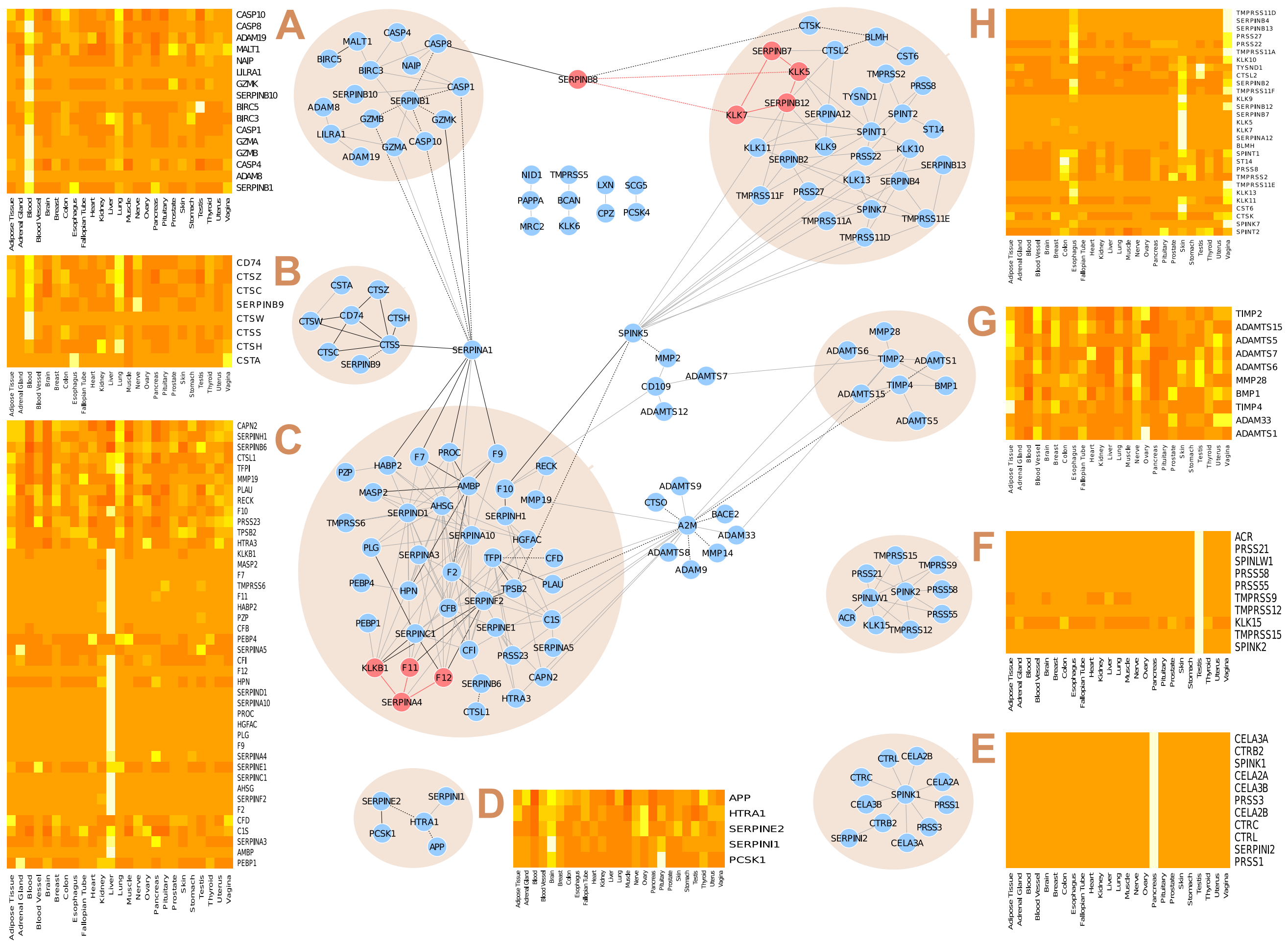

Figure S7. Coexpression of Predicted Inhibitor-Protease Pairs Shown as a Network

Proteases and inhibitors were connected if their coexpression (Spearman or Pearson correlation of expression values) was higher than 0.6 (GTEX_All_Max), and the inhibitor was annotated to inhibit a protease from the same family as the target or the protease was annotated inhibited by an inhibitor from the same family. Solid black lines show colocalization (proteins in the same compartment), black dashed lines anti-localization (one protein extracellular and the other in the cytosol or one in an organelle and the other in the cytosol) and solid grey lines missing annotation of one of the proteins. The resulting network is clustered (x- and y-axis are arbitrarily arranged to reflect clustering). Clusters are highlighted and labeled A-H. Heatmaps show normalized expression of genes in each cluster. Predictions (proteases, inhibitors, and their connections) selected for biochemical testing are highlighted in red.

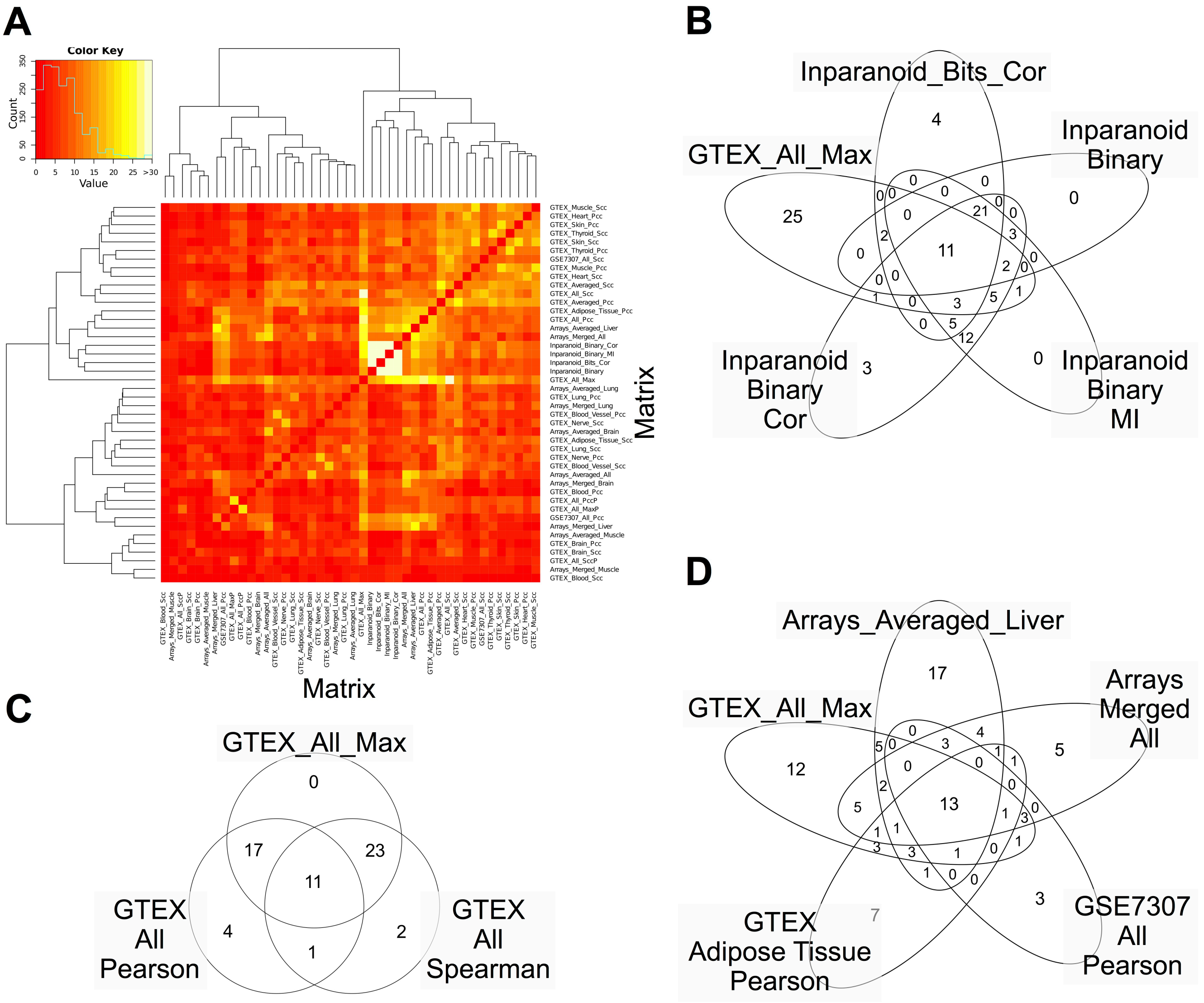

Figure S8. Overlap of Coexpression and Phylogenetic Similarity Matrices for Recovering Protease Web Inhibitions

Annotated true positive inhibitor-protease pairs found among the top 10% of pairs (predictions) from each matrix. (A) Heatmap of the overlap of recovered inhibitions. Values above 30 were capped. (B) Overlap of recovered inhibitions between GTEX_All_Max and Inparanoid matrices. (C) Overlap between various coexpression matrices. (D) Overlap of recovered pairs in GTEX-derived matrices using Spearman and Pearson correlations.

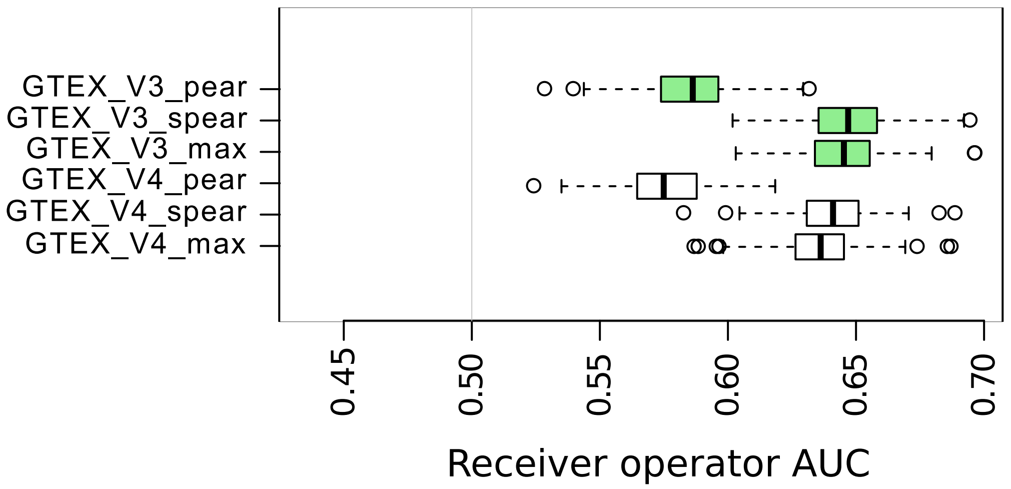

Figure S9: Prediction performance of coexpression matrices is unchanged in newer versions of GTEX.

Area under the curves (AUC) for the receiver operator characteristic of predictions of gold standard true positive and true negative inhibitory interactions based on two versions of GTEX: version 3 (V3, green) with 1660 and version 4 (V4, white) with 2921 samples. True negatives were subsamples to reflect the number of true positives (n=218). Coexpression matrices are calculated using Pearson (pear) and Spearman (spear) correlation and a method taking the maximum of the two (max).

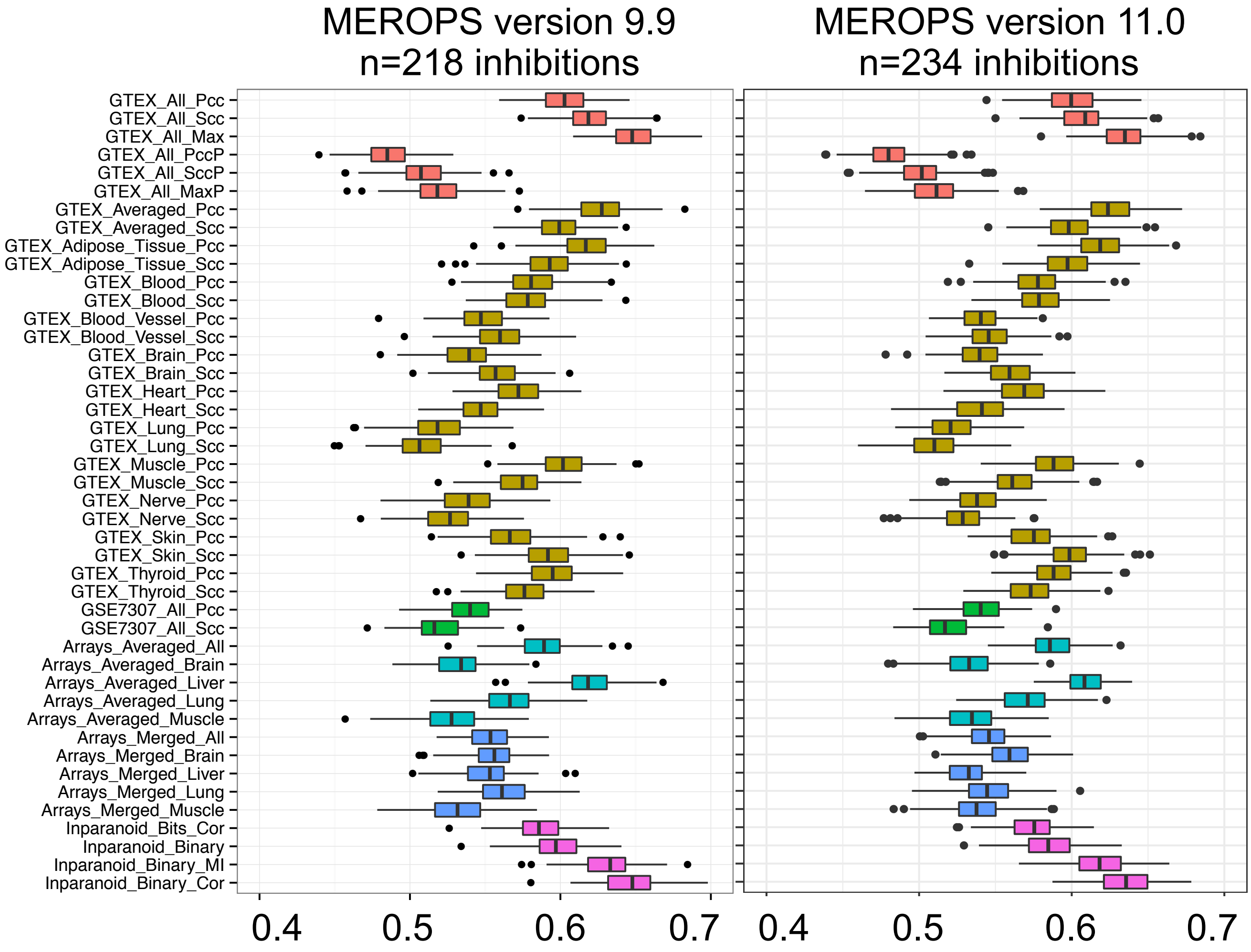

Figure S10: Prediction performance of coexpression matrices in MEROPS version 9.9 and 11.0

Area under the curves (AUC) for the receiver operator characteristic of predictions of gold standard true positive and true negative inhibitory interactions from MEROPS 9.9 and 11.0. True negatives were subsamples to reflect the number of true positives.

SUPPLEMENTARY REFERENCES

1. Mei, Y., Hahn, A. A., Hu, S., and Yang, X. (2011) The USP19 Deubiquitinase Regulates the Stability of c-IAP1 and c-IAP2. *J. Biol. Chem.* 286, 35380–35387

2. Sowa, M. E., Bennett, E. J., Gygi, S. P., and Harper, J. W. (2009) Defining the Human Deubiquitinating Enzyme Interaction Landscape. *Cell* 138, 389–403

3. Fang, Y., Mu, J., Ma, Y., Ma, D., Fu, D., and Shen, X. (2012) The interaction between ubiquitin C-terminal hydrolase 37 and glucose-regulated protein 78 in hepatocellular carcinoma. *Mol. Cell. Biochem.* 359, 59–66

4. Liu, J., Guo, Q., Chen, B., Yu, Y., Lu, H., and Li, Y.-Y. (2006) Cathepsin B and its interacting proteins, bikunin and TSRC1, correlate with TNF-induced apoptosis of ovarian cancer cells OV-90. *FEBS Lett.* 580, 245–250

5. Winnepenninckx, V., Lazar, V., Michiels, S., Dessen, P., Stas, M., Alonso, S. R., Avril, M.-F., Romero, P. L. O., Robert, T., Balacescu, O., Eggermont, A. M. M., Lenoir, G., Sarasin, A., Tursz, T., Oord, J. J. van den, and Spatz, A. (2006) Gene Expression Profiling of Primary Cutaneous Melanoma and Clinical Outcome. *J. Natl. Cancer Inst.* 98, 472–482

6. Ewing, R. M., Chu, P., Elisma, F., Li, H., Taylor, P., Climie, S., McBroom-Cerajewski, L., Robinson, M. D., O'Connor, L., Li, M., Taylor, R., Dharsee, M., Ho, Y., Heilbut, A., Moore, L., Zhang, S., Ornatsky, O., Bukhman, Y. V., Ethier, M., Sheng, Y., Vasilescu, J., Abu-Farha, M., Lambert, J.-P., Duewel, H. S., Stewart, I. I., Kuehl, B., Hogue, K., Colwill, K., Gladwish, K., Muskat, B., Kinach, R., Adams, S.-L., Moran, M. F., Morin, G. B., Topaloglou, T., and Figeys, D. (2007) Large-scale mapping of human protein-protein interactions by mass spectrometry. *Mol. Syst. Biol.* 3, 89

7. Yu, H., Tardivo, L., Tam, S., Weiner, E., Gebreab, F., Fan, C., Svrzikapa, N., Hirozane-Kishikawa, T., Rietman, E., Yang, X., Sahalie, J., Salehi-Ashtiani, K., Hao, T., Cusick, M. E., Hill, D. E., Roth, F. P., Braun, P., and Vidal, M. (2011) Next-generation sequencing to generate interactome datasets. *Nat. Methods* 8, 478–480

8. Micheau, O., and Tschopp, J. (2003) Induction of TNF Receptor I-Mediated Apoptosis via Two Sequential Signaling Complexes. *Cell* 114, 181–190

9. Schaefer, M. H., Fontaine, J.-F., Vinayagam, A., Porras, P., Wanker, E. E., and Andrade-Navarro, M. A. (2012) HIPPIE: Integrating Protein Interaction Networks with Experiment Based Quality Scores. *PLoS ONE* 7, e31826

10. Chatr-Aryamontri, A., Breitkreutz, B.-J., Oughtred, R., Boucher, L., Heinicke, S., Chen, D., Stark, C., Breitkreutz, A., Kolas, N., O'Donnell, L., Reguly, T., Nixon, J., Ramage, L., Winter, A., Sellam, A., Chang, C., Hirschman, J., Theesfeld, C., Rust, J., Livstone, M. S., Dolinski, K., and Tyers, M. (2015) The BioGRID interaction database: 2015 update. *Nucleic Acids Res.* 43, D470-478

11. Bossi, A., and Lehner, B. (2009) Tissue specificity and the human protein interaction network. *Mol. Syst. Biol.* 5, 260

12. Lonsdale, J., Thomas, J., Salvatore, M., Phillips, R., Lo, E., Shad, S., Hasz, R., Walters, G., Garcia, F., Young, N., Foster, B., Moser, M., Karasik, E., Gillard, B., Ramsey, K., Sullivan, S., Bridge, J., Magazine, H., Syron, J., Fleming, J., Siminoff, L., Traino, H., Mosavel, M., Barker, L., Jewell, S., Rohrer, D., Maxim, D., Filkins, D., Harbach, P., Cortadillo, E., Berghuis, B., Turner, L.,

Hudson, E., Feenstra, K., Sobin, L., Robb, J., Branton, P., Korzeniewski, G., Shive, C., Tabor, D., Qi, L., Groch, K., Nampally, S., Buia, S., Zimmerman, A., Smith, A., Burges, R., Robinson, K., Valentino, K., Bradbury, D., Cosentino, M., Diaz-Mayoral, N., Kennedy, M., Engel, T., Williams, P., Erickson, K., Ardlie, K., Winckler, W., Getz, G., DeLuca, D., MacArthur, D., Kellis, M., Thomson, A., Young, T., Gelfand, E., Donovan, M., Meng, Y., Grant, G., Mash, D., Marcus, Y., Basile, M., Liu, J., Zhu, J., Tu, Z., Cox, N. J., Nicolae, D. L., Gamazon, E. R., Im, H. K., Konkashbaev, A., Pritchard, J., Stevens, M., Flutre, T., Wen, X., Dermitzakis, E. T., Lappalainen, T., Guigo, R., Monlong, J., Sammeth, M., Koller, D., Battle, A., Mostafavi, S., McCarthy, M., Rivas, M., Maller, J., Rusyn, I., Nobel, A., Wright, F., Shabalin, A., Feolo, M., Sharopova, N., Sturcke, A., Paschal, J., Anderson, J. M., Wilder, E. L., Derr, L. K., Green, E. D., Struewing, J. P., Temple, G., Volpi, S., Boyer, J. T., Thomson, E. J., Guyer, M. S., Ng, C., Abdallah, A., Colantuoni, D., Insel, T. R., Koester, S. E., Little, A. R., Bender, P. K., Lehner, T., Yao, Y., Compton, C. C., Vaught, J. B., Sawyer, S., Lockhart, N. C., Demchok, J., and Moore, H. F. (2013) The Genotype-Tissue Expression (GTEx) project. *Nat. Genet.* 45, 580–585
